# From Individual Liquid Films to Macroscopic Foam Dynamics: A Comparison between Polymers and a Nonionic Surfactant

**DOI:** 10.1021/acs.langmuir.2c00900

**Published:** 2022-08-23

**Authors:** Alesya Mikhailovskaya, Emmanouil Chatzigiannakis, Damian Renggli, Jan Vermant, Cécile Monteux

**Affiliations:** † Soft Matter Science and Engineering, ESPCI Paris, CNRS, PSL University, Sorbonne University, 75005 Paris, Franceand; ‡ Institut de Chimie et des Matériaux Paris-Est, CNRS UMR 7182, 2-8 rue Henri Dunant, 94320 Thiais, France; § Department of Materials, ETH Zürich, Vladimir Prelog Weg 5, 8032 Zürich, Switzerland and; ∥ Polymer Technology Group, Eindhoven University of Technology, PO Box 513, 5600 MB Eindhoven, The Netherlands; # Soft Matter Science and Engineering, ESPCI Paris, CNRS, PSL University, Sorbonne University, 75005 Paris, France

## Abstract

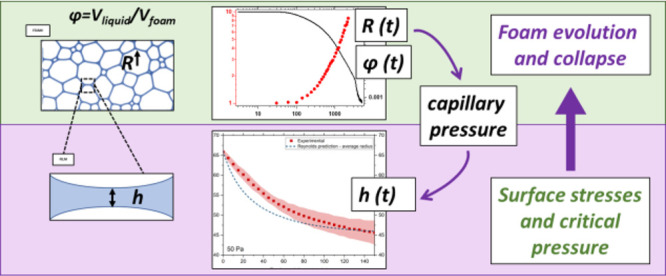

Foams can resist destabilizaton in ways that appear similar on a macroscopic scale, but the microscopic origins of the stability and the loss thereof can be quite diverse. Here, we compare both the macroscopic drainage and ultimate collapse of aqueous foams stabilized by either a partially hydrolyzed poly(vinyl alcohol) (PVA) or a nonionic low-molecular-weight surfactant (BrijO10) with the dynamics of individual thin films at the microscale. From this comparison, we gain significant insight regarding the effect of both surface stresses and intermolecular forces on macroscopic foam stability. Distinct regimes in the lifetime of the foams were observed. Drainage at early stages is controlled by the different stress-boundary conditions at the surfaces of the bubbles between the polymer and the surfactant. The stress-carrying capacity of PVA-stabilized interfaces is a result of the mutual contribution of Marangoni stresses and surface shear viscosity. In contrast, surface shear inviscidity and much weaker Marangoni stresses were observed for the nonionic surfactant surfaces, resulting in faster drainage times, both at the level of the single film and the macroscopic foam. At longer times, the PVA foams present a regime of homogeneous coalescence where isolated coalescence events are observed. This regime, which is observed only for PVA foams, occurs when the capillary pressure reaches the maximum disjoining pressure. A final regime is then observed for both systems where a fast coalescence front propagates from the top to the bottom of the foams. The critical liquid fractions and capillary pressures at which this regime is obtained are similar for both PVA and BrijO10 foams, which most likely indicates that collapse is related to a universal mechanism that seems unrelated to the stabilizer interfacial dynamics.

## I Introduction

Foams are multiphase materials consisting of gas bubbles dispersed in a continuous liquid phase. The liquid fraction ϕ, which is defined as ϕ = *V*_liquid_/*V*_foam_, plays a key role in controlling foam structure. For high values of ϕ, bubbles are merely suspended in the liquid phase, and the system presents rather a bubbly liquid. When the liquid fraction decreases below ϕ = 0.36, the bubbles jam, their arrangement becomes more compact, and they change their shape from spherical to polyhedral.^1^ Thin films formed at the contact area between bubbles meet in liquid channels called plateau borders (PBs), which, in turn, intersect in vertices, so that the liquid continuous phase forms an interconnected structure spanning the entire system.

Such a high specific surface area structure is thermodynamically unstable, because of the cost in surface energy. Typically, three mechanisms control the foam destabilization. Foam bubbles *coarsen* due to a difference in Laplace pressures across the foam, which drives diffusion of the gas from the smaller bubbles to bigger ones through the continuous phase. The bubbles can also *coalesce* when the thin liquid film between them ruptures. Finally, there is a macroscopic phase separation due to the difference in the gas and liquid densities that results in *drainage*. This last mechanism interferes in the foam aging as long as gravity is present. Therefore, there is always a tendency to change the liquid fraction distribution so that it is lower on the foam top and higher on its bottom part. All three mechanisms are inter-related,^2−4^ but usually drainage accelerates foam coarsening and bubble coalescence, due to the thinning of the thin liquid films.

Drainage is relatively well-described in the case of so-called “dry foams” with ϕ ≤ 0.1. One of the main assumptions in the theoretical description is that foams contain liquid mainly in PBs and vertices, whereas the amount of the liquid in the films can be neglected. The liquid flow in foams is dependent on the boundary conditions at air/liquid interfaces created by the adsorbed stabilizers. One distinguishes stress-free and stress-carrying interfaces depending on the magnitude of interfacial stresses, such as those related to surface viscoelasticity or Marangoni stresses (i.e., those related to surface tension gradients). The first situation of stress-free interfaces refers to the drainage dominated by fluid resistance in the vertices^5^ and the second one refers to those resistances being mainly found in the PBs.^6^ Both regimes have been observed in various experimental systems, together with the transition between them with the variation of the surface mobility.^7−9^ Nevertheless, literature reports identify clear deviations from these two regimes,^8,10−12^ which may be related to the role of the hitherto neglected thin liquid films, even in the limit of dry foams. Hence, it is worthwhile to pursue the link between the drainage at the scale of liquid films with the evolution of the macroscopic foams.^13,14^

Numerous studies have attempted to correlate the equilibrium properties of free-standing films (i.e., maximum disjoining pressure, equilibrium thickness) to foam stability^15−26^ to understand how phenomena that occur in the microscopic films affect the lifetime of macroscopic foams. However, the vast majority of these studies did not focus on the effect of surface stresses on foam stability and was thus able to provide, at best, a qualitative agreement between experiments on these two different length-scales.

Moreover, the comparison has been mostly limited to correlating the disjoining pressure of the thin liquid films (TLFs) to the overall foam lifetime, while, at the same time, acknowledging the fact that the intricate overall foam dynamics could not possibly be controlled by a single equilibrium film property. Thus, differences in the film rupture/bubble coalescence mechanisms,^4^ the unresolved quantification of interfacial viscoleastic stresses acting tangentially to the film and changing the hydrodynamic stresses, a possible blockage of the PBs by aggregates,^27^ and the limitations of the employed experimental techniques^4,28^ have all been suggested as possible reasons for these discrepancies, but have not been experimentally assessed. In the present work, we will specifically study how properties that can be assessed at the individual film level under dynamic conditions (disjoining pressure, interfacial stresses) can be related to certain events in the lifetime of the respective draining foams.

The choice of the model experimental systems is crucial in elucidating the underlying phenomena. Elimination of electrostatic interactions by using nonionic stabilizers can simplify the problem since for most cases the corresponding liquid films are stabilized only by short-range forces.^29−31^

In contrast to low-molecular-weight surfactants, amphiphilic polymers typically adsorb in layers with a thickness on the order of the gyration radius (*R*_g_) of the chains, with a small portion of the monomers anchoring the interface in trains, while the rest of the monomers form loops and tails.^32^ This conformation of adsorbed macromolecules provides a steric repulsion between the interfaces stabilizing the liquid films against their rupture. Solutions of amphiphilic polymers provide a great foam stabilizing effect even at relatively low concentrations where neither aggregation nor entanglement is observed, but systematic studies on these seem to be lacking. By studying these stabilizers, i.e., a nonionic surfactant and an amphiphilic polymer, with very different interfacial dynamics, we expect to probe systems with surface stresses of various origins so that we can observe to what extent the film and foam dynamics are different.

The experimental techniques should enable one to probe the drainage dynamics at the length scale of the liquid film and of the entire foam. The film stability is widely studied by the so-called thin film balance^33^ (TFB). However, most of the works so far have focused on equilibrium or slowly draining films without exploring the dynamics and the involved interplay of hydrodynamic forces with capillarity, interfacial stresses, and disjoining pressure.^34^ Specifically, previous work with the TFB was limited to slow, quasi-static drainage conditions under capillary pressures smaller than those typically developed in foams and was thus able to focus only on the interplay between surface stresses and disjoining pressure.

Modification of the classical setup with a pressure controller allows us to perform the measurements at driving pressures similar to those in the macroscopic foam drainage. We compare the results obtained on liquid films with the behavior of macroscopic foams stabilized either by a low-molecular-weight surfactant or by an amphiphilic polymer. Probing the foam drainage by the measurements of the foam conductivity evolution gives us direct access to the surface mobility. Combined with macroscopic foam visualization and microscopic bubble size determination, these measurements allow us to investigate the behavior of the foam and divide its lifetime into certain distinct regimes. The insight at the microscopic film level obtained by the dynamic TFB is then used to elucidate the possible physical mechanisms involved in these regimes of foam destabilization.

## II Materials and Methods

### II.A Materials

A nonionic surfactant, polyoxyethylene(10) oleyl ether (BrijO10 from Sigma–Aldrich) and an amphiphilic polymer, a partially hydrolyzed poly(vinyl alcohol), PVA (Mowiol 8-88, from Sigma–Aldrich), are used. The weight-average molecular weight (M_w_) of the PVA, as determined by gel permeation chromatography (GPC), was 63 500 ± 500 g/mol, and its polydispersity index is equal to 1.4. A vinylacetate (VAc) monomer content of 8% was determined by nuclear magnetic resonance (NMR) spectroscopy (see the Electronic Supporting Information (ESI)), which is slightly smaller than the 12% specified by the manufacturer. The distribution of VAc units was found to be slightly “blocky” with each VAc segment containing, on average, two monomers. The concentrations in foaming solutions (20 mM for BrijO10 and 0.1 wt % for PVA) are chosen such that the amount of surface active elements is the same for both systems, considering the fraction of acetate groups in PVA macromolecules that provide surface activity of the polymer. The concentration of BrijO10 is three orders of magnitude higher than its critical micelle concentration (CMC), which was reported to lie in the interval from 2.5 × 10^–5^ M to 4 × 10^–5^ M;^35,36^ these observations are in accordance with our data (see the ESI). Since BrijO10 and PVA do not carry any charge, we add 20 mM of sodium chloride into all foaming solutions to improve their conductivity response for the experiments on the liquid fraction evolution. The addition of NaCl at this concentration has no effect on the surface properties, both for PVA,^37,38^ as well as for Brij when its concentration is well above the CMC.^39^ To further confirm this, we also conducted TFB experiments both with and without NaCl for the two stabilizers and observed no difference on the measured film properties.

### II.B Time Dependence of the Surface Properties

The time-dependent evolution of the surface tension γ(*t*) was measured using an automated tensiometer (TRACKER, Teclis-Scientific) in the configuration of rising bubble. The experiments lasted 3 h, since the dynamics of polymer adsorption is rather slow. We measure the variation of effective interfacial tension during oscillation of interfacial bubble area *A* at a frequency *f* of 0.1 Hz and a surface deformation amplitude of 3%, which is related to an apparent surface compression modulus *K*_app_^′^:.

### II.C Bulk Viscosity

The bulk viscosity (η) of the foaming solutions was measured using a standard rotational rheometer (Model AR-G2 Rheometer, TA Instruments) using a cone–plate geometry with the cone angle of 2°, diameter of 40 mm, and truncation of 52 μm. Frequency sweeps performed in the range of 5–100 Hz ensure the Newtonian behavior of the foaming solutions. All measurements are made at 25 °C, and with a solvent trap to avoid evaporation. The viscosity data are presented in Table I.

**Table I:** Properties of the Stabilizer Molecules and Their Solutions: Molecular Weight of the Stabilizers (*M*_w_), Hydrodynamic Radius (*R*_H_), Solution Surface Tension (γ), Apparent Surface Elasticity , Surface Shear Viscosity (η_*s*_), and Bulk Viscosity (η)^a^

stabilizer	*M*_w_ (g/mol)	*R*_H_ (nm)	γ (N/m)	(N/m)	η_s_ (Pa s m)	η (Pa s)
BrijO10	709	6.4	31.3 × 10^–3^	1.2 × 10^–3^	<10^–7^	1.4 × 10^–3^
PVA	63 000	7.3	49.1 × 10^–3^	10.1 × 10^–3^	10^–6^	1.1 × 10^–3^

a Values for the interfacial characteristics correspond to a system age of 3 h.

### II.D NMR Spectroscopy

^1^H and ^13^C NMR spectroscopy was employed to determine the percentage of VAc units in the PVA and their distribution along the polymer chain. The measurements were conducted with a Brujer Avance IIID spectrometer at 25 °C. The samples were dissolved in D_2_O in 5 mm tubes. The ^1^H spectra were obtained at 500 MHz, while the ^13^C spectra at 125 MHz. The NMR spectra and the related discussion can be found in the ESI.

### II.E Surface Shear Rheology

The interfacial shear rheology was investigated with a custom built interfacial needle shear rheometer (ISR)^40^ that was based on the design of Brooks et al.^41^ and Reynaert et al.^42^ at *T* = 25 °C. Details can be found in the ESI.

### II.F Langmuir Trough Compression Measurements

Surface pressure–area “isotherms” of the PVA surfaces were measured in a rectangular Langmuir trough (internal area of 7.5 cm × 32.2 cm) (KSV-NIMA, Finland). Two different compression speeds were employed, namely, 2.5 and 10 mm/min. The surface pressure was measured with a Wilhelmy plate with a width of 19.62 mm and a thickness of 0.1 mm mounted on a balance (KSV Nima).

### II.G Dynamic Light Scattering

Dynamic light scattering (DLS) measurements were conducted with ALV CGS3 compact goniometer and a 22 mW HeNe laser light source at 25.0 °C and an angle of 90°. The micelles of BrijO10 were found to have a hydrodynamic radius, *R*_H_ equal to 6.4 nm; for PVA, it is 7.3 nm (average of three measurements).

### II.H Dynamic Thin Film Balance

**Figure 1:**
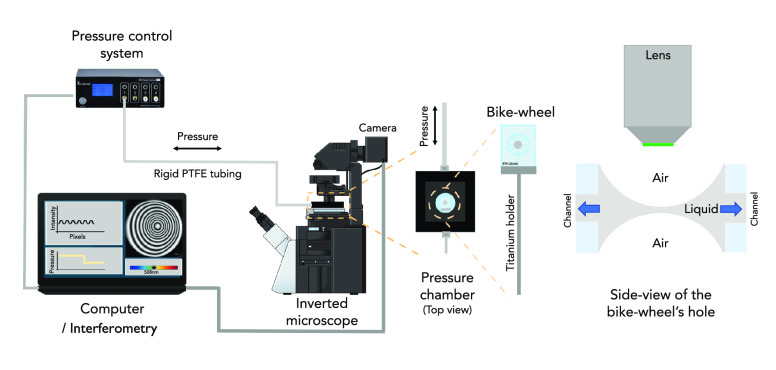
Sketch of the dynamic thin film balance setup and a zoom in of the cross section of the thin liquid film formed inside the bike-wheel’s hole. [Reproduced with permission from ref (50). Copyright 2020, Royal Society of Chemistry, London.]

The dynamic thin film balance technique (DTFB) is a microfluidic bikewheel device based on the initial design of Cascao-Perreira et al.^43^ Its main components are sketched in Figure 1 and have been described elsewhere.^44,45^ Thickness determination is done by interferometry, using Sheludko’s equation^33^ to calculate the equivalent thickness *h*_w_:
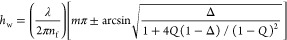
1where λ is the wavelength of the monochromatic light used, *n*_f_ and *n*_c_ are the refractive indices of the film and outer air phase, respectively, and *m* is the order of interference. Δ and *Q* are defined as Δ =  and *Q* = , where *I*_f_ is the intensity of the film and *I*_min_ and *I*_max_ are, respectively, the minimum and maximum intensities measured during the experiment. For planar films, this methodology results in a thickness resolution of ±2 nm. The refractive index of the solutions was assumed to be equal to that of water (*n*_f_ = 1.333) and thus eq 1 essentially allows the determination of the “equivalent film thickness”.^46^ For BrijO10 films that had an equilibrium thickness (*h*_eq_) close to 10 nm, a correction was applied considering the different refractive index of the surface layer,^47,48^ which allows the determination of the actual film thickness (see the ESI). Two different thicknesses are reported, depending on the area of the film in which we measured the intensity. The average thickness (*h*) corresponds to the average intensity as measured in the entire circular film region. In contrast, the thickness at the center (*h*_c_) corresponds to the average intensity of a smaller rectangular area of ∼100 pixels located at the film’s center. Image processing was done with ImageJ^49^ and Matlab. The effect of evaporation was minimized by adding excess solution in the pressure chamber, thus ensuring that the atmosphere is saturated. Samples were degassed in a recipient under vacuum to ensure that no dissolved air is present.

Two different types of experiments were conducted. First, the disjoining pressure of the films was evaluated using the classical equilibrium film method.^47^ The pressure was increased stepwise and the average thickness of the film was measured after an equilibration period of 10 min. The minimum pressure that can be applied when obtaining the disjoining pressure isotherm is set by the radius of the bike-wheel’s hole (*R*_bw_) and is equal to 2γ/*R*_bw_. Second, the drainage dynamics of the TLFs were assessed using the methodology of ref (45). A first pressure step of Δ*P* = 50 Pa was applied to ensure that the film was thinning slow enough for the Reynolds equation (eq 2) to be valid. For PVA an extra pressure step of Δ*P* = 200 Pa was applied to assess how surface stresses evolve with increased drainage velocity. The obtained drainage curves were compared to the prediction of the Reynolds equation:
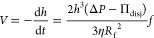
2where *V* is the thinning velocity, *R*_f_ the film’s radius, and *f* a mobility factor that describes deviations from the Poiseulle flow inside the film (for which *f* = 1). The Reynolds equation was solved numerically in Matlab with the Runge–Kutta method, using the experimentally determined disjoining pressure and the average experimental film radius in the regime where the film was planar. At least three measurements were conducted for both the drainage and the disjoining pressure measurements.

### II.I Foam Preparation

**Figure 2:**
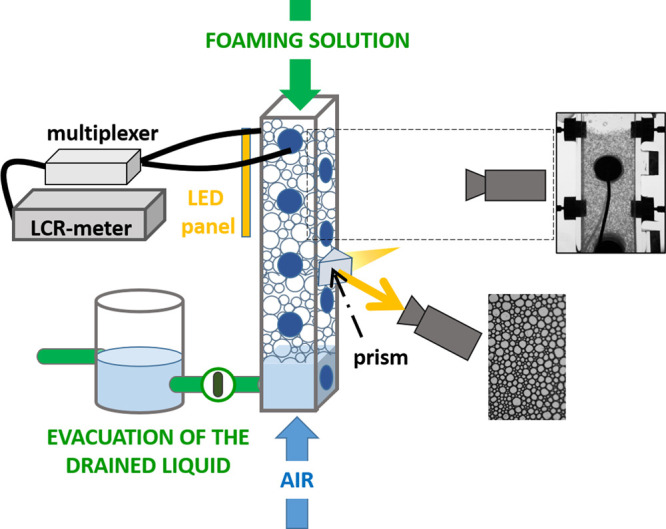
Experimental setup for studying of foam drainage. The foam is prepared by introducing air into the foaming solution. Simultaneously, a constant liquid flow from the top ensures a homogeneous distribution of the liquid fraction. The cell has electrodes to measure the foam conductivity, which gives the average liquid fraction at a fixed vertical position. Taking images at the cell surface using an optical prism gives the evolution of the average bubble size.

To create the foams, air is forced through a porous frit, localized at the bottom of an acrylic cell (225 mm height, 30 mm × 30 mm square cross section), covered by 50 mL of solution. During the foaming process, gravity induces drainage resulting in an inhomogeneous liquid fraction profile. To compensate the drainage flow, we continuously wet the foam from the top, similar to the process used by Carey and Stubenrauch,^9^ which involved injecting the foaming solution at a constant flow rate through four syringes arranged in the corners of the measuring cell. Such a configuration allows a uniform distribution of the liquid at the top of the foam without breaking of the bubbles in the upper layers. The liquid flow rate at this stage is up to *Q*_L_= 4 mL/min in the case of the slowly draining PVA-stabilized foam and *Q*_L_= 25 mL/min in the quickly draining BrijO10-stabilized foam, so that the produced wet foam displays moderate coarsening due to the increased thickness of the liquid films between the bubbles. The constant level of the liquid below the foam is assured due to a connection with a vessel containing a certain liquid volume. The excess of the drained liquid is evacuated from the system through a hole in the connected vessel. The setup is sketched in Figure 2. Note that, in such configurations, the measuring cell cannot be covered from the top and the upper layers of the bubbles are exposed to evaporation. The gas flow is switched off when the bubbles fill the cell from bottom to top. We then progressively slow down the liquid flow rate of the top injected foaming solution to decrease the value of the liquid fraction within the foam column to the values of 0.10–0.15. Once the desired homogeneous liquid fraction profile along the foam height is set, the liquid flow is stopped, and we let the foam drain freely. This moment is taken as the reference zero time *t*_0_ of the experiment.

### II.J Liquid Fraction Measurements

We obtain ϕ values from the foam electrical conductivity^51^ measured by pairs of circular electrodes, which have a radius of 4 mm (Figure 2). Six pairs of electrodes are evenly distributed from the top of the foam cell with the distance of 2.5 cm between the centers of electrodes. An additional pair of electrodes is located close to the bottom of the cell: it remains covered with the foaming solution and measures the reference conductivity allowing to retrieve the value of ϕ. The electrodes are connected to an impedance meter (LCR Meter, Chroma 11021) operating at a frequency of 1 kHz and a voltage of 1 V. The apparatus measures the resistance of a parallel resistor–capacitor equivalent circuit, the value of which is reciprocal to conductivity.

### II.K Bubble Size Measurement

The initial bubble radius *R*_init_ is controlled by the size of the pores and the surface tension of the foaming solution.^52^ We measure it straight after bubble formation by imaging a thin layer of foam using a microscope.^53^ We find that *R*_init_(BrijO10) = 60.5 μm and *R*_init_(PVA) = 79 μm. The preparation of the foam takes 30–45 min, so that the average bubble size evolves during this time due to the foam coarsening. To get the average bubble size at *t*_0_, defined as the starting point of free drainage, and monitor its time evolution, we take pictures of the bubbles at the surface of the measuring cell through a prism attached to the cell wall (see the dashed zone in Figure 2). Using an open source image processing program ImageJ, we employ the protocol described in ref (54) and determine the surface area *A*_b_ of bubbles before converting it into the bubble radii *R*(*t*) = . The value *R*(0) corresponds to *t*_0_. The Sauter mean radius, which is defined as ⟨*R*(*t*)⟩ = , averaged over *n* bubbles at the image, increases during the foam aging. We find that *R*(0)_PVA_ = 156 μm and *R*(0)_BrijO10_ = 335 μm. Being different in absolute values, the *R*(0)_PVA_ and *R*(0)_BrijO10_ remain in the interval for submillimetric bubbles, which are commonly used in studies of foam drainage.^7−9,55^ Since the size of the analyzed image is restricted by the perimeter of the prism, *n* decreases with time. We perform the analysis only for *n* > 100.

The distribution of the bubble sizes is analyzed by calculating the probability density function (PDF) at a given foam age as , where *V*(*R*_*i*_ < *R* < *R*_*i*_ + Δ) is the total volume of the bubble with the radius *R* between *R*_*i*_ and *R*_*i*_ + Δ, *V*_tot_ the total volume of the bubbles, and Δ the bin size of the histogram.

## III Results and Discussion

### III.A Thin Film Stability

The evolution of the thickness profile during drainage for specific pressure steps, the time scales for breakup and the disjoining pressure of the films were all investigated using the DTFB. The drainage experiments allow a quantification of effects of changes in the stress-boundary conditions and provide insight on the role of hydrodynamics. The applied pressure step of Δ*P* = 50 Pa, combined with the bikewheel’s Laplace pressure 2γ/*R*_bw_, resulted in a total driving pressure that is of the same magnitude but somewhat smaller than the Laplace pressure, which drives the drainage in the actual foams (which is evolving with time up to ∼400 Pa). The thin film measurements can allow us to decouple the effects of surface stresses in foam drainage from other phenomena, such as coalescence and coarsening.

#### III.A.1 Disjoining Pressure

**Figure 3:**
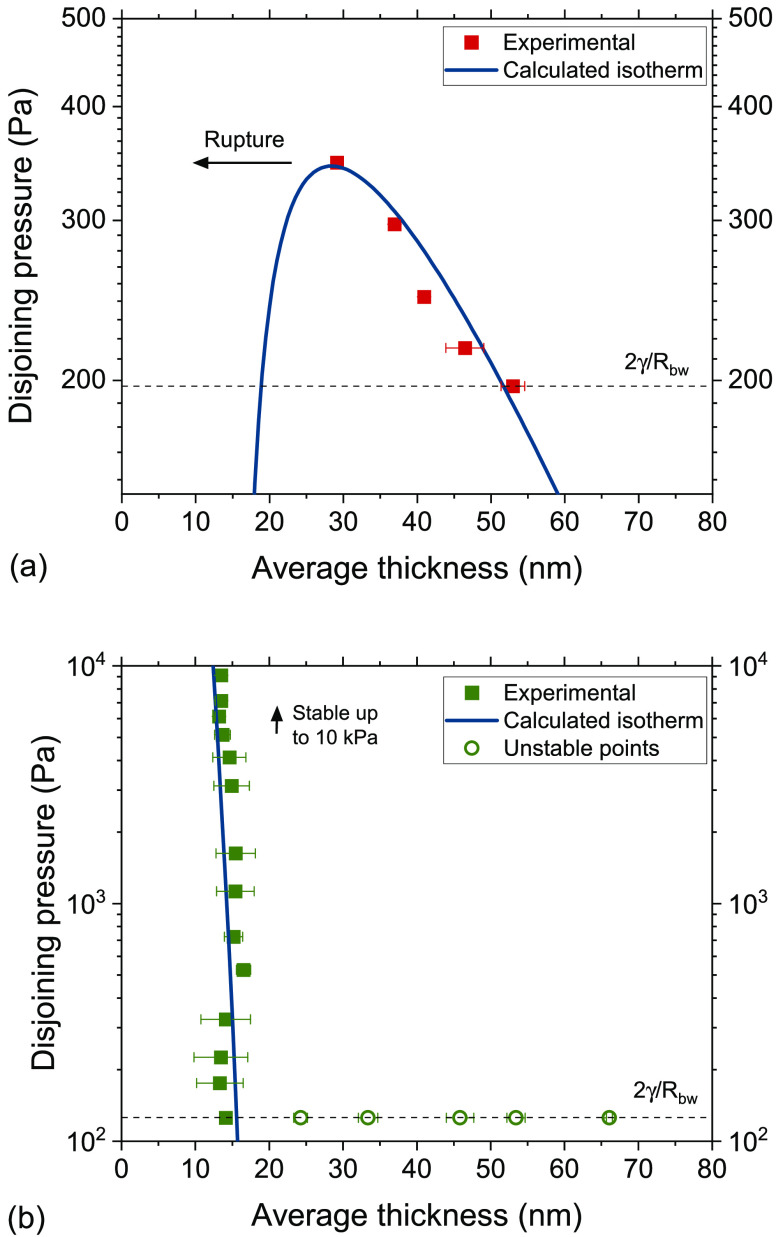
Disjoining pressure isotherms of (a) PVA and (b) BrijO10. The calculated disjoining pressure isotherms are shown with the solid blue lines. The static Laplace pressure in the bikewheel is noted by a dotted line.

The disjoining pressure isotherms of the PVA and the BrijO10 solutions are shown in Figures 3a and 3b, respectively. The films of PVA were stable at an average thickness slightly higher than 50 nm. Increasing the applied pressure resulted in an exponential decrease in thickness, in agreement to previous studies on film stabilized by PVA with various molecular characteristics.^56−58^ The films became unstable and ruptured at a critical pressure of 350 Pa. The experimental disjoining pressure is the sum of two contributions, namely of the steric interactions between the adsorbed PVA chain segments (Π_st_) and of the DLVO attractive van der Waals (vdW) interactions (Π_vW_):
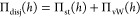
3

The calculated Π_disj_ is shown with a solid blue line in Figure 3a. The steric interactions were modeled following Semenov et al.,^59^ using a modified model by Mondain-Moval et al.:^58^
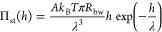
4where *k*_B_ is the Boltzmann constant, *T* the temperature, *R*_bw_ the radius of the bike-wheel’s cell, *A* a fitting parameter that is dependent on the radius of the film and the adsorption density, and λ a fitting parameter known as the decaying length, i.e., the distance at which two opposing chain segments start to interact. The contribution of the interactions between planar films is equal to
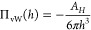
5where *A*_H_ is the nonretarded Hamaker constant, which was calculated based on the Lifshitz theory^60^ and found to be 3.7 × 10^–20^ J. Adsorbed polymers are known to affect the vdW interactions between opposing surfaces.^60^ Because of the steep decrease of the polymer volume fraction along the *z*-direction,^61^ the similar dielectric properties of the polymer solution with the aqueous core,^62,63^ and the large thickness of the film, the change in the vdW forces due to the polymer brush, and thus the actual location of the interface, has a negligible effect on the determined λ. An exact calculation of the Π_vW_ with and without the adsorbed PVA, as well as the estimated effect of vdW interactions on λ can be found in the ESI.

Apart from the trend in the Π_disj_(*h*), eq 3 is also able to predict the critical pressure (*P*_crit_) at which the vdW interactions dominate, resulting in film rupture. Similar values for *P*_crit_ were also reported by Espert et al.^57^ on a randomly distributed PVA/VAc copolymer. A decaying length λ of 17.8 nm provided the best fit. This value is in agreement with existing studies on free-standing PVA-stabilized films,^57^ as well as on PVA layers adsorbed on solid surfaces^64−69^ (ESI). Small differences can be attributed to the fact that the decaying length is dependent on the distribution of VAc units, the *R*_g_ of the polymer, the polymer–solvent interactions, the surface concentration and the applied pressure.^32,56,57^

Steric effects between adsorbed (co)polymers are usually described by a scaling model of de Gennes,^70^ which considers brush–brush interactions. In our case, the model of Semenov et al.^59^ was found to describe Π_disj_(*h*) better, suggesting that interactions occur due to the longer dangling chain ends, in agreement to the relatively large decaying length of λ ≈ 2*R*_g_ (ESI).

The disjoining pressure of BrijO10 is a sum of two contributions. The vdW forces remain present but now a structural oscillatory force occurs, which is due to the structuring of micelles:
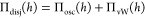
6

The disjoining pressure with an oscillatory force can be described by the model of Trokhymchuk et al.:^71^
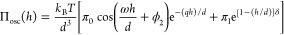
7where *d* is the diameter of the object giving rise to the structural forces (assumed to be equal to 2*R*_H_, as determined by dynamic light scattering (DLS)), *h* is the thickness of the film, ϕ is the initial volume fraction of the micelles (ϕ = 0.142), and the remaining terms (apart from *k*_B_*T*) are fitting parameters. The first term in the bracket accounts for the repulsive structural, and attractive depletion component of the Π_*osc*_. For *h* < *d*, no micelles are present in the film. The exponential term of eq 7 describes the steric repulsion between two adsorbed surfactant layers.

The experimental (symbols) and predicted (solid lines) disjoining pressure isotherms are shown in Figure 3b. The oscillatory forces were found to be smaller than the Laplace pressure exerted by the curvature of the bike-wheel’s hole (2γ/*R*_*bw*_, shown as a dotted line on the figure) and thus they were not observed. Similarly, eqs 6 and 7 predict a negligible structural contribution to Π_disj_. Basheva et al.^39^ measured the disjoining pressure of a similar Brij surfactant and observed structural forces with a maximum pressure of ∼1000 Pa. However, (i) the Brij that they investigated has a smaller micelle size, (ii) the concentrations that they employed were larger, and (iii) the radius of the cell’s hole was larger. Since all these parameters should bring the oscillatory forces into the experimentally observable pressure window,^72^ their absence in our system is rather expected. The thickness transitions that were unstable in our experiments (as *P*_max_ < 2γ/*R*_bw_) are shown in Figure 3b with open symbols. They correspond to thickness differences of Δ*h* ≃ 2*R*_H_, suggesting the expulsion of a single micellar layer (see the ESI). This observation, which also has previously been made for the case of other nonionic surfactants,^73,74^ is in contrast to the variable Δ*h* systematically reported for ionic surfactants.^75^ Regardless of the small Π_osc_, the Newton black film (NBF) that was formed at *h* < *d* was stable and did not break, even at the maximum pressure that can be applied in our setup (∼10 kPa). This is again in agreement with results on films stabilized by similar nonionic surfactants.^39^ The final thickness of the film was ∼10 nm, in agreement to the results of Maruganathan et al.^76^ on similar surfactants. The length of two fully extended BrijO10 molecules is ∼8.7 nm (based on the length of the bonds; see the ESI), which indicates that some water probably remains in the film regardless of the magnitude of the applied pressure.^76^ As it will be discussed in III.B.2, the different film stabilities at equilibrium are in general agreement with the evolution of liquid fraction observed in the macroscopic foam.

#### III.A.2 Film Drainage

The overall stability of the thin liquid films is not only controlled by the disjoining pressure, but also by the surface stresses that oppose the outflow of water. When it comes to macroscopic foam stability, the surface stresses might even be more important at the early stages of foam lifetime,^77,78^ when the thickness of these interstitial films in the foam are usually much larger than 100 nm, and thus the effect of disjoining pressure is negligible. The quasi-static drainage of films, which are then assumed to remain planar and of constant radius can be described by a generalized Reynolds equation,^34,79,80^ as indicated above in eq 2.

**Figure 4:**
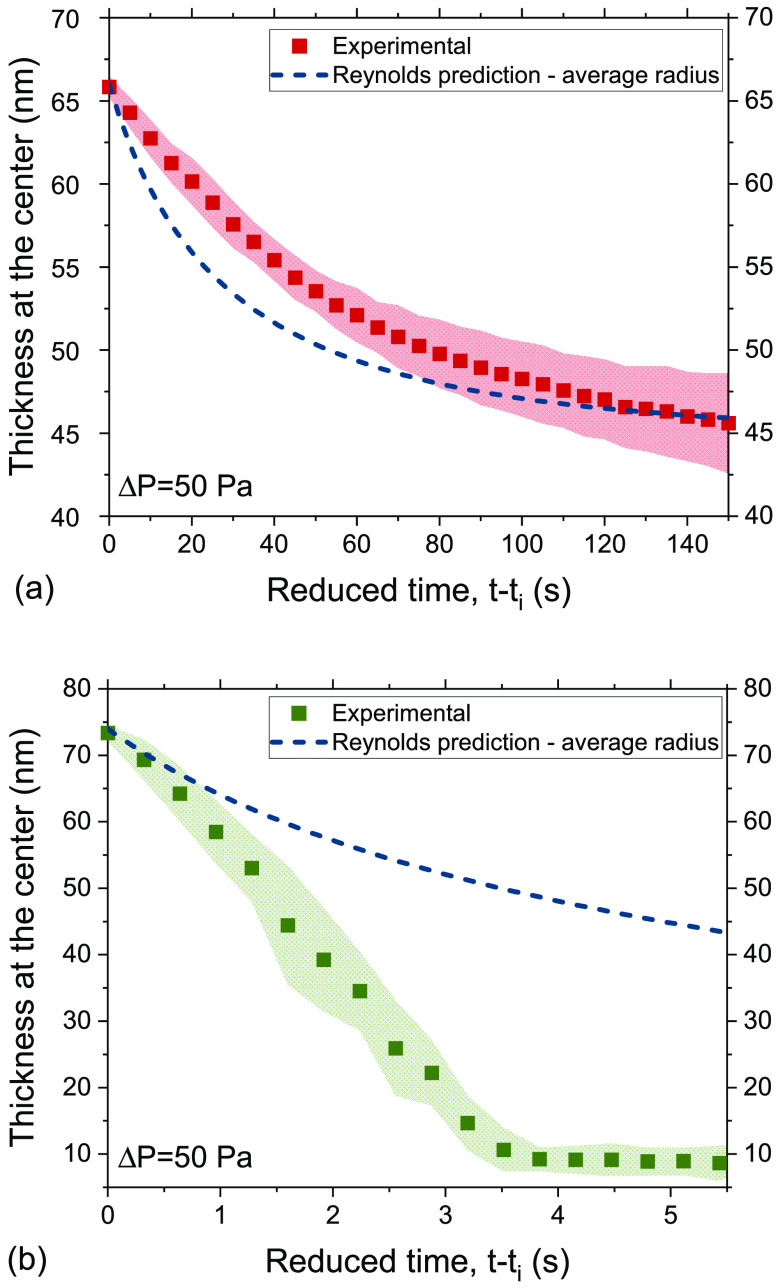
Experimentally observed and quasi-static limiting (eq 2 for *f* = 1) drainage curves of (a) PVA and (b) BrijO10 films. The average radii of the films were 0.17 and 0.07 mm, respectively. *t*_*i*_ is the time at which the film planar film forms.

**Figure 5:**
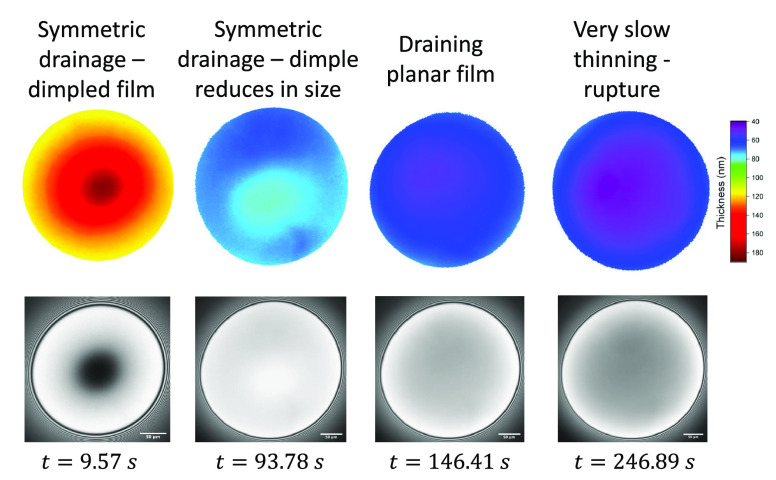
Microinterferometry images and the corresponding 3D plots of a PVA film at different stages of drainage for a pressure jump of 50 Pa. Initially, a dimple is formed (as can be seen from the nonuniform intensity). Then, a symmetrical drainage of a planar film is observed until rupture.

The experimental drainage curve of PVA for Δ*P* = 50 Pa (as an average of three measurements) (Video S1) is shown in Figure 4a, together with the prediction of eq 2 for *f* = 1. Since eq 2 is only valid for planar films, *t*_*i*_ is the time at which the small dimple, which initially forms, gets completely smoothed out and a planar film is formed (see Figure 5). The film thinned slowly for a drainage time of more than 150 s. The agreement of the experimental trends with the predictions with eq 2 for *f* = 1 indicates that the surfaces of PVA were stress-carrying to the extent they are immobile, in agreement to the observations of macroscopic foam drainage at small *t* (Figure 7).

There are two main contributions to the surface stresses of the PVA films during drainage. As the interface is being strained, surface rheological and Marangoni stresses both contribute to the total stress carriage of the surfaces.^34^ The Boussinesq number (*Bq*), which describes the interplay between surface shear and bulk viscosity in foam and film drainage, is written as *Bq* = η_s_/(η*R*_f_). A surface shear viscosity of η_s_ ≈ 10^–6^ Pa s m of the PVA-stabilized air/water interface was measured with the ISR (Figure S1 in the ESI), which results in *Bq* ≈ O(10) using η ∼ 10^–3^ Pa s and *R*_f_ ≈ 10^–4^ m. Although a value of *Bq* ≈ O(10) indicates that, indeed, the surface shear viscosity contributes partially to the total surface stress carriage, both simulations and experiments have shown that higher *Bq* numbers are typically needed to achieve Poiseuille flow with zero surface velocity inside the film^81−85^ that was observed in our drainage measurements (Figure 4a). Similarly, at *Bq* ≈ 10, the flow in the PBs of foams occurs faster than what would be expected from Poiseuille flow.^7,86−88^

The shapes of the films during drainage also indicate a highly stress-carrying surface (Figure 5). At low Δ*P*, the drainage of the films was symmetric, with the dimple that was initially formed at the film’s center gradually draining until a thick planar film is formed. The symmetric drainage and the absence of Marangoni-caused instabilities, such as the dimple wash-outs and the thickness corrugations, is generally related to the stabilizing effect of surface viscosity^89^ and elasticity.^84^

However, the observation that the effects of surface shear viscosity alone does not account for the high stress-carrying capacity of the PVA-stabilized films, indicates that the contributions of surface dilatational viscoelasticity and/or of Marangoni stresses are non-negligible. The apparent dilatational moduli  values obtained by the drop shape analysis (DSA) method are an order of magnitude higher for the PVA-stabilized air/water interface than for BrijO10 (recall Table I). The obtained apparent moduli are dependent not only on the transport of surface-active species from and at the interface but also on the inherent rheological properties of the interface. Although these two contributions can only be fully decoupled by elastometry,^90,91^ various factors indicate that Marangoni stresses, which are expected to show up at PVA surfaces of low polymer concentration,^92^ dominate the drainage of the PVA films and, therefore, also of the foams:

- Langmuir compression isotherms at different speeds were observed to be only marginally different, with the maximum surface pressure being only ∼3 mN/m (see the ESI), in agreement with previous literature results.^93^ Homogeneous compressional deformations are hence not expected to induce significant stresses.
- Yet, clear clues are the drainage of the films at Δ*P* = 200 Pa > 2γ/*R*_bw_, which becomes asymmetric and inhomogeneous, with the dimple slowly moving toward the rim of the film (ESI and Video S3). This is typical of a Marangoni-driven instability,^89^ which, in the PVA films, occurs slowly, most likely because of the small contribution of surface viscosity.
- The surface tension of PVA solutions close to the studied concentration of 0.1 wt % indeed show relatively large variations with concentration (ESI), which would entail that small spatial variations in concentration lead to significant gradients and strong enough Marangoni stresses.
- The bulk and surface diffusion constants of PVA are 1–3 orders of magnitude smaller than those of soluble low-M_w_ surfactants.^35,94−96^ Thus, for a given Δ*P*, the resulting surface concentration gradients can be expected to be higher.

In congruence with the observations made here of a planar drainage with a stress-carrying interface, a Poiseuille flow inside the thin films has been observed in polymer-stabilized emulsion films^97^ and was explained based on the two-region flow model of brushes.^98^ In this model, it is assumed that the outer layer of the adsorbed brush “protects” the inner layer through a hydrodynamic screening mechanism. Although the higher surface viscosity and , might essentially reflect the same physical origin with this effect, i.e., the irreversible adsorption of the PVA chains in train, loop, and dangling end conformations, with each segment interacting with the neighboring ones,^32,99^ our results rather suggest that the traditional contributions of Marangoni stresses and surface viscosity suffice to explain the observed drainage behavior and there is no need to invoke a hydrodynamic screening effect.

Interestingly, all PVA-stabilized films that were measured ruptured despite the fact that the applied Δ*P* was smaller than the maximum disjoining pressure (350 Pa) (Figure 3a). This is probably related to surface concentration gradients that are caused by the fast drainage, that change locally the magnitude of the steric repulsive forces.

**Figure 6:**
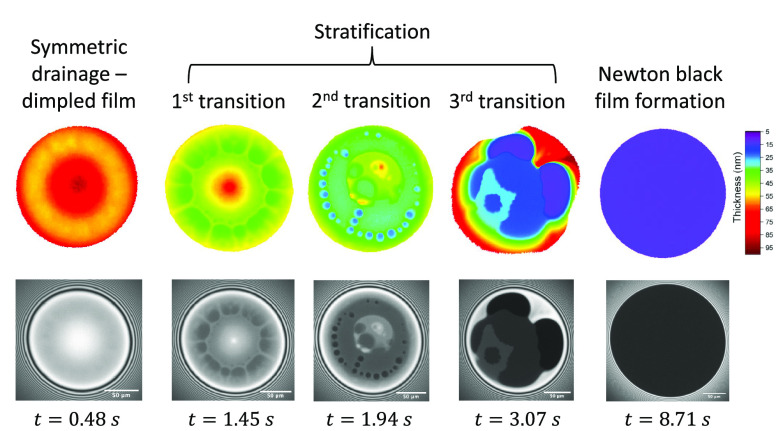
Microinterferometry images and the corresponding 3D plots of a BrijO10 film at different stages of drainage for a pressure jump of 50 Pa. Again a dimple forms, but it now becomes unstable when the first dark domains expand. Stratification is then observed until an equilibrium NBF is formed.

In contrast to PVA, the BrijO10 films drained much faster than what would be expected from a stress-carrying boundary condition and a resulting Poiseuille flow in the thin films (Figure 4b) (Video S2). First, the drainage of the films down to the equilibrium thickness of a NBF occurred in ∼4 s, 2 orders of magnitude faster compared to the PVA films. Second, the films showed stratification, thickness corrugations and dimple-washouts (Figure 6). The two last effects are expected in films with Marangoni stresses,^89,94,100^ while the first one is a result of the structuring of micelles inside the film.^47^ The fact that the deviations from Poseuille flow become larger as the film thins (Figure 4b) is usually an indication that surface and bulk diffusion oppose the development of Marangoni stresses.^79^

Soluble surfactants often show surface shear inviscidity.^101^ Indeed, no surface shear viscosity could be measured within the operational window of the ISR, resulting in a *Bq* ≪ 1. Surface stresses in the BrijO10 films are thus expected to depend solely on the surface tension gradients and the resulting Marangoni stresses. The latter, however, are seemingly not strong enough to ensure a Poiseuille flow inside the film, which is in agreement to previous studies on films stabilized by these types of surfactants close to CMC.^82,85,94,102−105^

In the following section, we will consider the macroscopic evolution of the aqueous foams stabilized by PVA or BrijO10, and its correlation with the stability of thin liquid films.

### III.B Foam Drainage and Collapse

**Figure 7:**
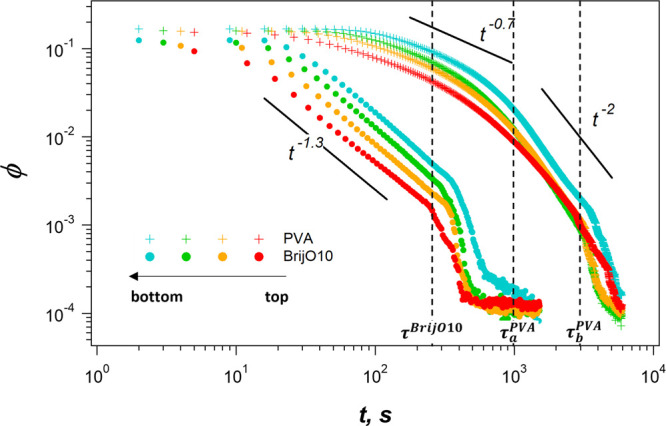
Evolution of the liquid fraction as a function of time at different vertical positions in the foam stabilized by PVA (crosses) or by BrijO10 (circles).

The experiments are such that free-draining foams stabilized with a PVA or with BrijO10 are observed macroscopically. Figure 7 shows the time evolution of the liquid fraction ϕ(*t*) along the column for foams initially prepared with a homogeneous ϕ-distribution. In both cases, the liquid fraction decreases over time with a drainage front propagating from the top to the bottom. Following Carrier et al.,^10^ we estimate the distribution of the liquid in the studied foams between the films and the PBs (ESI) and show that drainage occurs primarily due to the liquid flow in the latter ones. Two distinct regimes are observed for BrijO10, whereas there are three different regimes in the case of PVA. In the first regime, for short times, the liquid fraction varies as a power law with time, ϕ ∝ *t*^β^ for both systems with a different exponent β for BrijO10 and PVA. The underlying reason for the observed exponent values are discussed later in Section III.B.1. After a time τ^BrijO10^ ≈ 200 s, the liquid fraction decreases abruptly, because of the propagation of the foam rupture front. For PVA, the same collapsing front is observed, only at much longer times, τ_b_^PVA^ ≈ 3000 s. In addition, for PVA, an intermediate regime is observed for times between τ_a_^PVA^ ≈ 1000 s and τ_b_^PVA^ ≈ 3000 s, during which the liquid fraction decreases in an accelerated manner, because of isolated bubble coalescence events, while the overall foam volume remains constant. In the following, we discuss the short and long time behavior separately. We relate the liquid fraction evolution rate with the bubble growth in both systems, since the latter can induce a transition in foam permeability for the liquid flow and, therefore, impact the foam drainage rate.^5,6,8^

#### III.B.1 Short Time Drainage Behavior

At short times, for *t* < τ^BrijO10^ = 300 s, the liquid fraction of the BrijO10-stabilized foam gradually decreases with time as φ ∝  with β^BrijO10^ ≈ −1.3. As discussed previously in the literature,^5,8,9^ the expression −2 < β < −1 typically corresponds to a pluglike flow regime for stress-free interfaces and is consistent with the observed fast drainage of the individual thin liquid films with low surface stress carriage presented above. It is typical for low-molecular-weight surfactants since their fast adsorption–desorption dynamics and high diffusion coefficient do not allow the development of significant Marangoni stresses.

**Figure 8:**
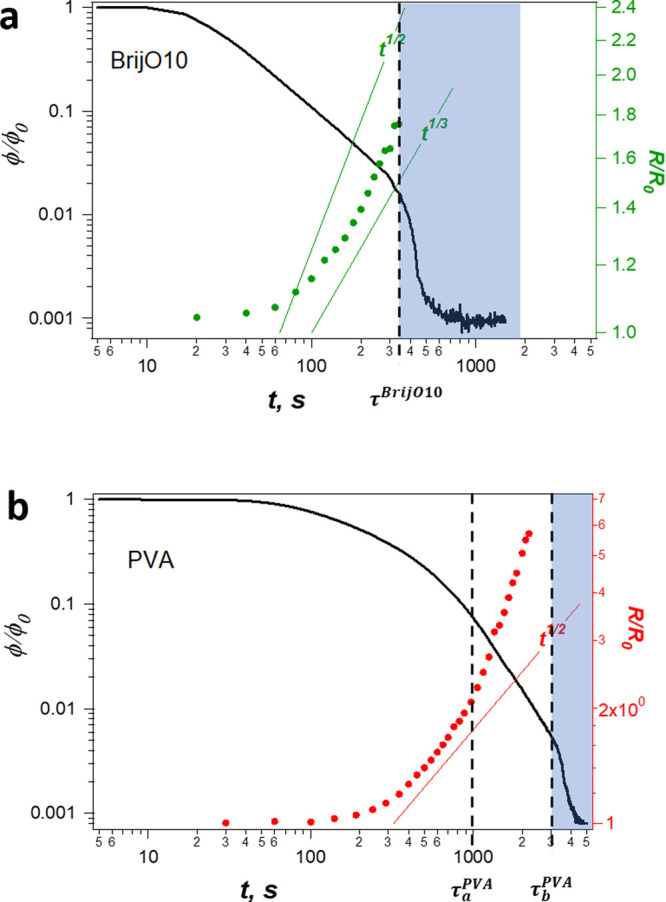
Evolution of the average bubble radius (symbols) and the liquid fraction (line) at the vertical position *Z* = 150 mm for different vertical positions in the foam stabilized by (a) BrijO10 and (b) PVA. The shaded zone corresponds to the collapse front propagation time period.

In Figure 8a, the time dependence of the average bubble size retrieved from the images taken at the surface of the sample cell for the BrijO10 foam is plotted together with the evolution of the liquid fraction at corresponding vertical positions. For a coarsening foam, it is predicted that the average bubble size initially grows in an exponential manner and then as a power law in a so-called “self-similar regime”, where the bubble radius grows as *t*^1/2^ for dry foams and *t*^1/3^ for sufficiently wet foams.^1,3,106^ The bubble size polydispersity evolves and reaches a constant value of 48% in the self-similar regime.^107^ Consistently, one can see in Figure 8a that, for the BrijO10-stabilized foam, the evolution approaches the *t*^1/2^-scaling as it becomes drier due to the drainage. Thus, to summarize, the evolution of BrijO10-stabilized foam at initial stages is governed by the drainage within the liquid network of PBs and films with low stress carriage surfaces and the bubble size growth is caused by coarsening.

For the PVA solution at short times for *t* < τ_a_^PVA^, the rate of drainage scales as φ ∝ , with  ≈ −0.7, which lies in the interval −1 < β < −^2^/_3_ determined for the Poiseuille-like flow in the case of stress-carrying surfaces.^5,8,9^ This observation is consistent with the slow drainage of the individual PVA films reported above. We also estimate *Bq* =  with the radius of PBs (*r*_PB_) in the PVA foams calculated using the following expression:^5^
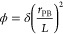
8based on the Kelvin cell model with geometrical parameter δ ≅ 0.17 and the PB length *L* = *D*_b_/2.7, where *D*_b_ is the bubble diameter. To obtain *r*_PB_ from eq 8, we use the bubble size and the liquid fraction ϕ obtained experimentally and presented in Figure 8b. *Bq* evolves over time as the foam ages, but it remains of the order of 10 (see the ESI), which is consistent with the value calculated for the thin film experiment. Since the values of η_s_ and η are the same for both experiments, the reason for this is that the size of the films in the DTFB and the radius of the PBs are of the same order of magnitude (i.e., 100 μm). As for the films, we note that the observed slow drainage cannot be related only to the effect of the surface shear viscosity because it is not sufficient for immobilization of the interfaces, and their stress-carrying character should originate from Marangoni stresses developed in the adsorbed layers of PVA macromolecules.

#### III.B.2 Long-Time Behavior

##### Intermediate Regime in PVA Foams for : Homogeneous Bubble Coalescence

III.B.2.a

**Figure 9:**
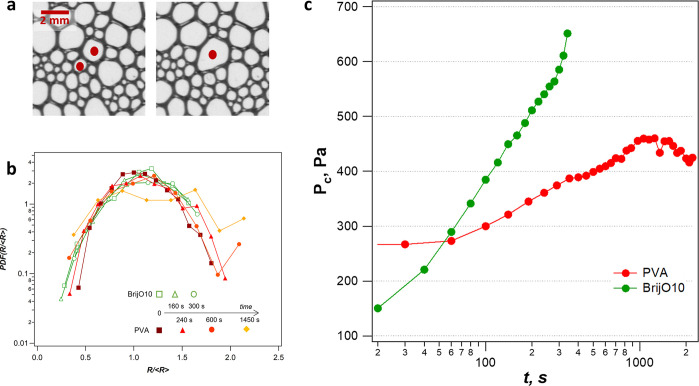
(A) Sequence of images taken at the cell wall for PVA-stabilized foam at the age of ∼1500 s; (b) probability distribution functions ((PDFs) for BrijO10-stabilized foams (open symbols) and PVA-stabilized foams (solid symbols) at different system ages; (c) capillary pressure in the PVA and BrijO10 foams estimated from values of *r*_PB_ calculated from eq 8 and using the experimentally measured values of liquid fraction and bubble size.

For the PVA-stabilized foam, at *t* = τ_a_^PVA^ ≈ 1000 s, the bubble size growth accelerates, as shown in Figure 8b. The average bubble radius deviates from the *t*^1/2^ behavior expected for the coarsening of dry foams. This acceleration in bubble size growth indicates that coarsening cannot be the only mechanism at play and that the foam ages also due to coalescence. This is confirmed from the sequence of images where bubbles can be observed to merge from time to time (Figure 9a). The frequency of coalescence events detected at the images is quite low, because of a restricted area of observation. We note that this coalescence process proceeds while the foam volume remains constant and seems to occur in a homogeneous manner throughout the entire foam.

In Figure 9b, we present the evolution of the probability density function  for the PVA foam, as well as for BrijO10 foam as a comparison. For the PVA-stabilized foam, the peak flattens during the aging due to bigger bubbles resulting from coalescence events. In contrast, the bubble size distribution of the BrijO10-stabilized foam does not change with time, since it is expected for self-similar regime in a coarsening foam.^1^

One can estimate the value of the capillary pressure *P*_c_ = γ/*r*_PB_ developed in the foam films at this stage from the values of ϕ and the bubble size shown in Figure 9a and using eq 8. We observe that this coalescence-induced bubble growth starts when ϕ = 0.01 and R = 355 μm, corresponding to values of the radius of the PBs (*r*_PB_ = 65 μm) and capillary pressures of the order of 450 Pa (Figure 9c). This is in very good agreement with the values of the critical disjoining pressure obtained using the DTFB. Monin et al.^25^ also reported such a homogeneous collapse that was proved to be governed by the behavior of the NBF. The authors suggested that an increase of the surface viscosity leads to a better resistance of the thin films to thickness fluctuations and consequently to a slower foam collapse at the critical pressure. Similarly, in our case, it seems that the critical pressure is reached and that the stress-carrying PVA layers can stabilize the thin films against strong fluctuations leading to a more homogeneous and slow foam collapse.

The acceleration of the growth in bubble radius rate observed after 1000 s coincides with a faster drainage regime, as shown in Figure 8b. Several studies have discussed the coupling between foam drainage and bubble size evolution caused by coarsening.^2,8^ Indeed, as the bubble size increases, the size of the PBs get bigger, and one may expect a decrease of *Bq* and a subsequent increase of foam permeability, hence, in the drainage regime.^7^ As shown in the ESI, we calculate *Bq* from the radius of the PBs obtained from eq 8 and using the experimentally measured liquid fractions and bubble size. Surprisingly, we find that *Bq* is constant over the course of the experiment, because of the mutual compensation of the liquid fraction decrease and the bubble size growth (see eq 8) and therefore cannot account for a permeability variation in the foams. We note that, in our experiments, the bubble size remains <1 mm, a size for which anomalous variations of the permeability with the bubble size were observed.^7,8,10^ A possible effect is that the bubble coalescence results in an increase of foam polydispersity as the fraction of larger-sized bubbles becomes more important. In Figure 9b, one can see an increase of the number of large bubbles over time. Yazhgur et al.^55^ showed that the big bubbles control the drainage rate in a foam, even if their number is small. Indeed, by increasing locally the permeability, they create preferential paths for the liquid flow, and therefore, they determine the drainage regime.

##### III.B.2.b Collapsing Front for *t* > τ^Brij^ and *t* > τ_b_^PVA^

After ∼3000 s for PVA foams and 200 s for BrijO10 foams, a rupture front is observed to propagate rapidly from the top of the foam to the bottom. When this collapse front passes at the level of an electrode pair at a fixed position, the foam no longer covers the electrode surface completely. Therefore, the resulting electric conductivity corresponds to that of the wetting layer on the electrodes. The resulting change in the slope of the liquid fraction evolution (shaded zone in Figure 8) presents a reliable indicator for the stage of the foam collapse.

Such a destruction is often observed in aqueous foams^1,25,108,109^ and starts with coalescence occurring in the top layers of bubbles with liquid films thinned out because of drainage. As the foam gets drier and the radius of the PBs reduces in diameter, *P*_c_ increases over time. For BrijO10, the foam collapse is observed when *P*_c_ reaches 500 Pa. This capillary pressure is well below the critical disjoining pressure, which could not even be probed in the experimental range of the DTFB (Figure 3b) and, hence, was estimated to be >10 kPa. Such a discrepancy between the critical capillary pressure measured for isolated films and foams has already been observed for foams stabilized by low-molecular-weight surfactants.^4,25,109−111^ Interestingly, this front collapse is also observed in the case of the PVA foams, right after the slow coalescence regime described earlier. To the best of our knowledge, this is the first time that these two types of coalescence regimes have been reported successively for a given system.

Several mechanisms have been suggested in the literature to explain this sudden foam collapse.^18,19,25,110,111^ Although understanding the physical mechanism controlling the front collapse of the foams at long times is beyond the scope of this study, there are two main remarks that can be made, based on our experimental observations.

First, the fast coalescence regime is observed for both PVA and BrijO10 foams at times of *O*(10^3^) s, much beyond the time scales associated with the thin film drainage. Therefore, it can be concluded film drainage is not the rate-determining step in this collapse process.

Second, the critical liquid fractions and critical capillary pressures at which we observe the front collapse are similar for both PVA and BrijO10, i.e., on the order of 10^–3^ and 500 Pa, respectively, although both systems are very different. The diffusion coefficient of PVA and BrijO10 differ by almost an order of magnitude, which seems to rule out any influence of the diffusion and adsorption dynamics on this phenomenon.^110^

## IV Conclusions

The drainage and collapse of foams stabilized by either a partially hydrolyzed PVA or by a nonionic surfactant (BrijO10) was studied using time-resolved macroscopic measurements of the liquid fraction and the bubble sizes and compared to the microscopic dynamic and equilibrium properties of isolated films as studied with a dynamic thin film balance (DTFB). By comparing at the same capillary pressure, we were able to observe remarkable quantitative agreement between experiments.

The stress-boundary condition was shown to be the same in both foams and films. The PVA-stabilized surfaces were rendered stress-carrying by both the surface shear viscosity and the Marangoni effect. This resulted in slow drainage both at the foam and the film level. In contrast, the surfaces of BrijO10 have less stress-carrying capacity. because of a weaker Marangoni effect and, thus, drainage at both length scales proceeded much faster due to plug-flow-like conditions.

We estimated the capillary pressure in the foams from the liquid fraction and the bubble size and showed that the occurrence of isolated coalescence events between bubbles in the PVA foam closely matched the maximum disjoining pressure due to steric interactions that the films can withstand.

The homogeneous coalescence in the PVA foams was followed by a front propagation. The critical liquid fraction for the onset of this instability was found to be of the same order of magnitude for both PVA and BrijO10. Although the mechanism underlying the instability is still to be understood, the fact that it is observed for both stabilizers, despite their inherently different interfacial dynamics and stress-carrying capacities, indicates that foam collapse is probably related to a universal mechanism. More experiments with high enough spatiotemporal resolution are needed to confirm this hypothesis.

Typically, agreement between experimental results on single foam films and macroscopic foams is limited to a qualitative level.^1,4,27^ However, we show here that quantitative agreement can be achieved if the experiments are conducted at similar capillary pressures and probe the same phases of the foam and film lifetimes. The dynamic and equilibrium properties of free-standing TLFs, as studied by the DTFB, can thus provide clear insights into the dominating resistances against drainage, coarsening, and coalescence in foams and can be correlated to specific processes during the lifetime of the latter.

## Supporting Information Available

Interfacial shear rheometry of the PVA interfaces; compression “isotherms” of the PVA interfaces; correction of the thickness of the Brij thin films; supplemental results (drainage and disjoining pressure) on foam films; NMR and GPC results; supplemental results on foams; surface tension isotherms (PDF)
Drainage of a PVA-stabilized film at Δ*P* = 50 Pa. The film drains symmetrically until it reaches its equilibrium constant thickness (Speed 5×) (AVI)
Drainage of a BrijO10-stabilized film at Δ*P* = 50 Pa. The film shows stratification until it reaches its equilibrium constant thickness (Speed 1×) (AVI)
Drainage of a PVA-stabilized film at Δ*P* = 200 Pa. The film shows asymmetric drainage until it ruptures (Speed 1×) (AVI)

## Supplementary Material

la2c00900_si_001.pdf

la2c00900_si_002.avi

la2c00900_si_003.avi

la2c00900_si_004.avi
