# Supplementary material for: From Individual Liquid Films to Macroscopic Foam Dynamics: A Comparison between Polymers and a Nonionic Surfactant
Source: Langmuir. 2022 Aug 23;38(35):10768–80. doi: 10.1021/acs.langmuir.2c00900 (PMC9454262; doi:10.1021/acs.langmuir.2c00900)
Supplement: Supplementary file 1 — la2c00900_si_001.pdf [file la2c00900_si_001.pdf]

From individual liquid films to macroscopic foam dynamics: a comparison between polymers and a non-ionic surfactant

Alesya Mikhailovskaya,[1,2,*] Emmanouil Chatzigiannakis,[3,4,*]
Damian Renggli,[3] Jan Vermant,[3] and Cécile Monteux[1,†]

[1]*Soft Matter Science and Engineering, ESPCI Paris, CNRS,*
PSL University, Sorbonne University, 75005 Paris, France

[2]*Institut de Chimie et des Matériaux Paris-Est,*
CNRS UMR 7182, 2-8 rue Henri Dunant, 94320 Thiais, France

[3]*Department of Materials, ETH Zürich,*
Vladimir Prelog Weg 5, 8032 Zürich, Switzerland

[4]*Polymer Technology Group, Eindhoven University of Technology,*
PO Box 513, 5600 MB, Eindhoven, The Netherlands[‡]

[*] These two authors contributed equally.

[†] cecile.monteux@espci.fr

[‡] e.c.chatzigiannakis@tue.nl

S I. INTERFACIAL SHEAR VISCOSITY OF PVA

The interfacial shear rheology of 0.1 % PVA shown in Fig. S1 was investigated with a custom built interfacial needle shear rheometer (ISR) based on the design of Brooks *et al.* [1] and Reynaert *et al.* [2] at $T = 25\,°\mathrm{C}$. Here, a brief overview of the device and the measuring technique is presented, a detailed description can be found in Renggli *et al.* [3].

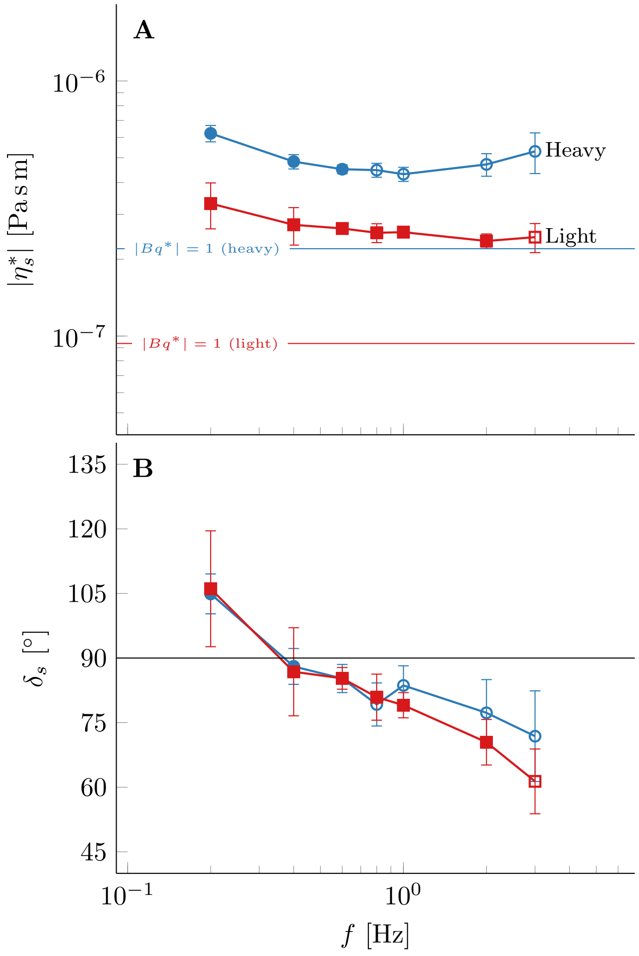

Fig. S 1. **Interfacial shear rheology of PVA:** (A) Norm of the complex interfacial shear viscosity and (B) phase angle of the corresponding complex interfacial modulus of 0.1 % PVA at a water–air interface at 25 °C with $\eta = 1.1\,\mathrm{mPa\,s}$. The properties of the two magnetic needles are described in Table S.1. Lines of $Bq = 1$ are indicated for both probes. Open and closed symbols reach a relative error of 1×10^{-5} and 1×10^{-10} during the iterative subphase correction, respectively. Errorbars are calculated based on primary data as explained in Ref.[3].

The complex interfacial shear modulus η_s^* is very small but still measurable with the ISR

($|Bq^*| = 2-3$). The scattering of the phase angles δ_s and its decrease with increasing frequency might be a consequence of the very small viscosity. Furthermore, the calibration of the two needles was performed with a separate interface. The calibrated instrument compliance depends not only on the inhomogeneity of the magnetic field, but also on interactions between the needle and channel wall due to a non-ideal wetting behaviour. Therefore, the instrument compliance could be different during the PVA measurements.

Sample preparation and experimental procedure

50 mL of 0.1 % PVA in water was added to a crystallizing dish, which was cleaned beforehand with acetone, ethanol, and milli-Q water. A glass channel with width of 12 mm was rinsed thoroughly with acetone, ethanol, and milli-Q water, plasma cleaned, and placed in the center of the crystallizing dish such that the water–air interface level equals half the channel height. Then, the magnetic probe was rinsed with milli-Q water and added to the channel. Frequency sweeps with two different magnetic needles were performed, where the first three of 13 acquired oscillating periods were discarded. The physical properties of the two magnetic needles can be found in Table SS.1.

Setup

Two magnetic coils were positioned in Helmholtz configuration and powered by two power supplies. A constant base current of 0.75 A was applied trough each coil to position the magnetic probe and modulated by a function generator in an anti-Helmholtz fashion. The two current signals were acquired by measuring the voltages across a resistors of 0.94 Ω placed in series to each coil with an acquisition rate of 50 Hz. The two current signals were subtracted from each other resulting in the driving current $I(t) = (V_1 - V_2)/R = I_0 \sin(\omega t - \delta_I)$.

The position of the probe was acquired by a CMOS camera at a rate of 25 Hz. The images were processed in real time with NI vision and the edge of the needle is tracked resulting in the position signal $z(t) = z_0 \sin(\omega t - \delta_z)$. The camera was mounted on an inverted microscope equipped with a 4× Plan objective. To reduce noise from vibrations and air flows, the ISR is enclosed by a Plexiglas box and placed on an active vibration isolation table.

Data processing

The discrete Fourier transforms of the current $I(t)$ and position $z(t)$ signals were performed with LabVIEW and the resulting amplitude ratio I_0/z_0 (units [A/pixel]) and phase angle difference were calculated. The subphase correction described by Verwijlen *et al.* [4] is performed using a finite difference method to decouple the interfacial from the bulk flow. The implemented codes are available for download (`https://softmat.mat.ethz.ch/opensource.html`). A subphase density of $0.997\,\mathrm{g\,cm^{-3}}$ and subphase viscosity of $1.1\,\mathrm{mPa\,s}$ were used.

Calibration

The instrument compliance k^{-1} and the force constant C_{I-F} relating the applied current I with the force F acting on the magnetic probe as

$$F = C_{I-F} I \quad , \tag{S.1}$$

are measured with a calibration procedure as proposed in Ref. [5] on a clean milli-Q–air interface. A subphase density of $0.997\,\mathrm{g\,cm^{-3}}$ and subphase viscosity of $0.894\,\mathrm{mPa\,s}$ were used. The quantified k and C_{I-F} are shown in Table S.1.

TABLE S.1. **Properties of the magnetic probes for the ISR:** l, r and m are the length, radius and mass of the needle, respectively. k^{-1} is the instrument compliance and C_{I-F} the force constant translating the input current to interfacial stress. k and C_{I-F} result from the calibration. The channel with is 12 mm.

	l [mm]	r [µm]	m [mg]	k [$\mathrm{N\,m^{-1}}$]	C_{I-F} [$\mathrm{N\,A^{-1}}$]
Heavy	23.5	200	5.8	3.5×10^{-5}	4.5×10^{-6}
Light	26.0	85	1.6	8.8×10^{-6}	2.3×10^{-7}

S II. COMPRESSION "ISOTHERMS" OF PVA

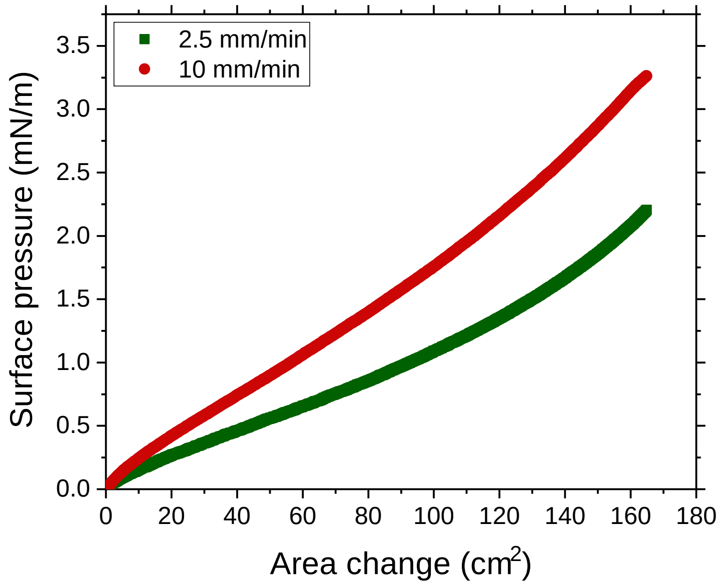

Fig. S 2. **Compression "isotherms" of PVA:** Surface pressure as a function of area change for two different barrier speeds.

S III. CORRECTION OF THE THICKNESS OF BRIJO10 FILMS

For films with $h_w \sim 10$ nm, the equivalent thickness of the film determined by Sheludko's equation (Eq. 1 in main article) has to be corrected according to the three-layer model [6]:

$$h = h_w - 2h_{tail}\left(\frac{{n_{tail}}^2 - n^2}{n^2 - 1}\right) - 2h_{head}\left(\frac{{n_{head}}^2 - n^2}{n^2 - 1}\right) \tag{S.2}$$

where h_{tail} and h_{head} are the lengths of the hydrophobic tail and the hydrophilic head of the surfactant, and n_{tail} and n_{head} the respective refractive indices. For BrijO10 $h_{tail} = 2.43$ nm and $h_{head} = 1.9$ nm (calculated using the formula of Tanford [7]). Following the usual practice [8, 9], the refractive indices were assumed to be approximately equal to those of decaethyleneglycol ($n_{head} \simeq 1.47$ [10]) and octadecane ($n_{tail} \simeq 1.44$ [11]). This procedure results in a decrease in measured thickness equal to 3.7 nm. For the PVA films, the application of such a correction is impossible as the refractive index of the interface is a function of z following the decrease in polymer concentration as we move away from the a/w surface [12]. Moreover, the actual length of the hydrophilic and hydrophobic segments is unknown. Nevertheless, Van Vliet estimated that the error for not applying this correction for PVA films is 0.6% [13].

S IV. SUPPLEMENTARY RESULTS ON FOAM FILMS

A. PVA films

1. *Estimation of decay length*

Knowing the molecular characteristics of the employed PVA/VAc copolymer, as obtained by the NMR and the GPC experiments, we can estimate the expected value of the decaying length λ at which opposing adsorbed chains start interacting. Given the assumptions needed to makes such an estimation, the values of λ obtained here are very approximate and only indicative of whether the experimental decaying length value is realistic or not.

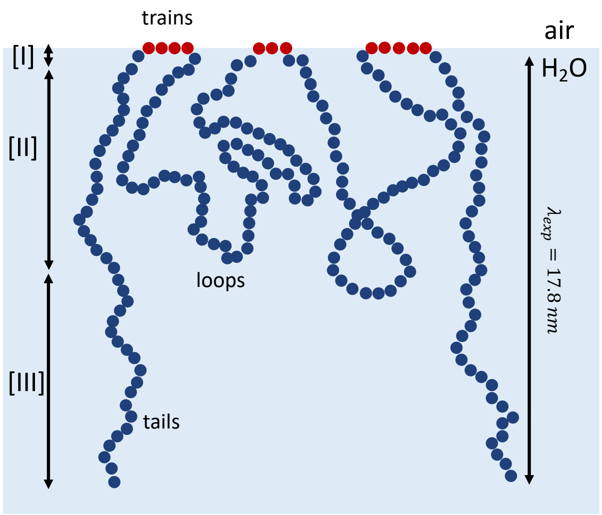

Fig. S 3. **An adsorbed PVA/VAc chain:** An adsorbed chain containing of trains, loops and tails. Red circles correspond to the hydrophobic VAc units while blue circles to the hydrophilic VA units. Regimes [I], [II], and [III] correspond to proximal, central, and distal region respectively [14].

The employed PVA has weight-average molecular weight of 63548 g/mol (GPC) and an 12% of VAc units as obtained by ^{1}H NMR and 8% obtained by ^{13}C NMR. The molecular weight of a VA and a VAc unit is 43.05 and 86.09 g/mol, respectively. Thus, each PVA/VAc chain consists of 1090 VA units and 47 VAc units. On average the VAc units are distributed along the polymer chain in duplets (^{13}C NMR). Thus, there are in total approximately $N_{VAc} = 24$ VAc train segments per chain, dividing the chain into 23 loops and 2 danging ends. Here we assume that the VAc trains are distributed equally along the chain, dividing it into loops and dangling ends of equal

length (Fig. S.3). This is of course a very crude approximation. The average number of VA monomers per loop (or danging end) segments is 44. Given that the length of a VA unit is 0.25 nm [15], we calculate an approximate length for the dangling VA ends equal to 11 nm, similar to the experimental value of $\lambda = 17.8$ nm. The observed deviation between the calculated and the experimental value is probably due to: (i) the distance between VAc train segments is not equal as assumed here, but rather random as expected, (ii) some of the VAc segments might be in the bulk film and not adsorbed [16], and (iii) the the studied polymer is not monodisperse.

2. *Comparison with other studies*

The value of $\lambda \simeq 18$ nm that we determined experimentally is similar to the values for the decaying length that been reported by other researchers [17–22] for PVA. In Fig. S.4 we plot our experimental disjoining pressure isotherm together with the results of Espert *et al.* [18] and Lyklema and Van Vliet [17] on foam films stabilized by PVAs of different molecular weight, but at similar low concentrations.

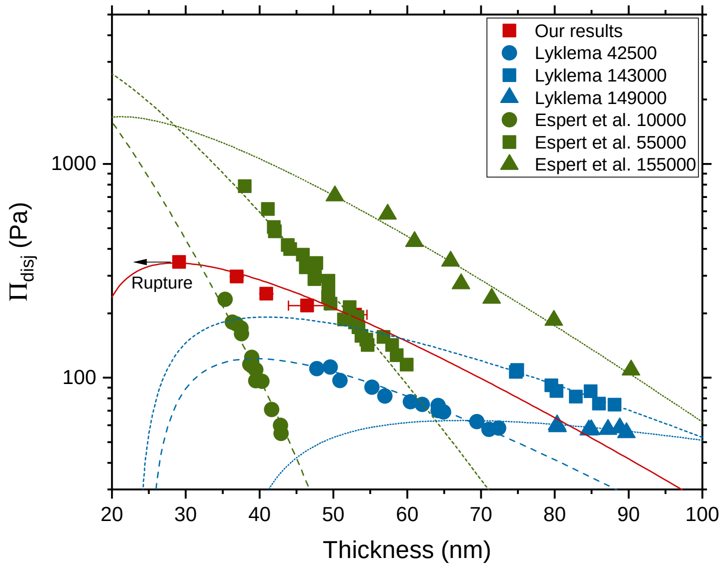

Fig. S 4. **Disjoining pressure isotherms of various PVAs:** The disjoining pressure isotherm of our study plotted together with the results of previous studies [17, 18]. The isotherms, which were calculated with the method explained in the main text, are shown as lines. Details on the studies and the obtained λ are shown in Table S2.

Although Espert *et al.* [18] determined using the same fitting procedure the decaying lengths for

the PVAs that they studied, they did not consider the contribution of the attractive vdW interactions to the total disjoining pressure. Attractive vdW interactions affect slightly the obtained λ in the case of foam films, but are negligible for emulsion films as the Hamaker constant is an order of magnitude smaller. We refitted their results following the procedure mentioned in the main article and applied it to the results of Lyklema and van Vliet [17] as well. In Ref. [17] the continuous phase was not water, but glycerol. Using a refractive index of 1.434 [23] and a relative permittivity of ~ 43 [24] a non-retarded Hamaker constant of $6.69 \cdot 10^{-20}$ J/mol was calculated.

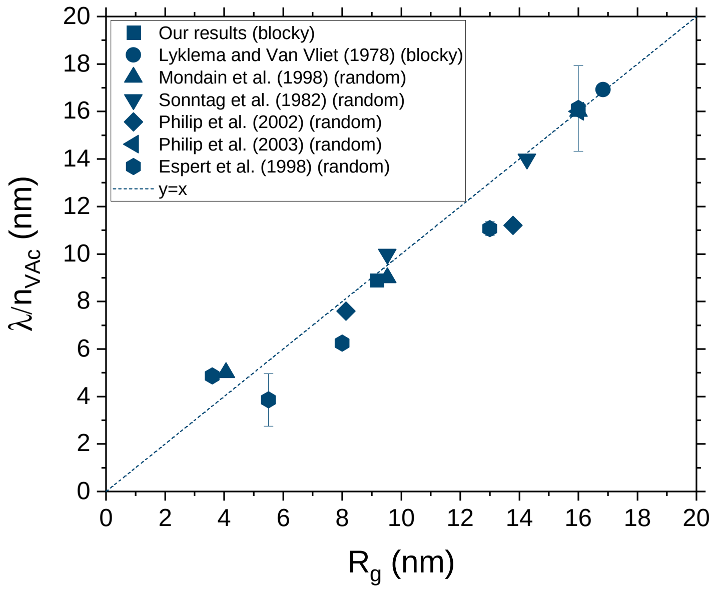

Fig. S 5. **Decay length:** The scaled decay lengths reported by various studies employing PVA as a function of the radius of gyration. Details about the studied systems and the determination of λ and R_g for each study can be found in the main text.

The experimental and fitted disjoining pressure isotherms are shown in Fig. S.4, while the molecular characteristics of the employed polymers and the obtained λ are shown in Table S2. It is evident that a random distribution of VAc units as studied by Espert *et al.* [18] results in much lower decaying lengths compared to those with a “blocky” distribution of VAc for similar molecular weight and VAc content. The dependence of steric interactions on the type of VAc distribution has already been suggested by Van Vliet [13, 17]. In Fig. S. 5 we plot the λ as obtained by the procedure described above, together with the reported values in other studies [18, 21, 22] as a funtion of the radius of gyration of the polymer. Sonntag *et al.* [19] reported the interaction energy exerted by adsorbed PVA on quartz surfaces as a function of thickness. The

results were reconverted to disjoining pressure using [25] $\Pi_{disj} = -dG/dh$ (where G is the free energy of the film) and were then fitted with Eq. 4 of the main article. Therefore, the obtained λ for Sonntag *et al.* entail a great deal of uncertainty. The radius of gyration (R_g) was calculated following Graessley [26]:

$$R_g = \sqrt{\frac{b^2 C_\infty M_w}{3 M_u}} \tag{S.3}$$

where $b = 0.154$ nm is the C-C bond length, $C_\infty = 8.9$ [27] is the characteristic ratio, and $M_u = 43.053$ g/mol is the molecular weight of the repeating unit. For the PVA used in our study, an $R_g \simeq 9$ nm was calcualated. The ratio R_H/R_g is approximately 0.8, as expected for linear polymers in good solvents [28] and specifically for PVA in water [29].

To confirm that indeed the reason for the different dependence of the decaying length on the thickness is the distribution of VAc units along the PVA chain, we divide λ by the number of VAc units per hydrophobic segment block. Given that the VAc content of all the PVAs studied is similar, all data overlap (Fig. S.5). The linear dependence $\lambda \sim R_g$ agrees with theory [30, 31] and experiment [18, 32].

3. *Effect of the van der Waals interactions on the determined decaying length λ*

Adsorbed polymers are known to affect the vdW interactions between opposing interfaces as they, locally at least, change the dielectric properties of the thin film [35].

The volume fraction, and thus also the dielectric properties, of the polymer chains decreases significantly as we move away from the air/liquid interface [36]. A precise calculation of the vdW interactions is non-trivial, as it would require treating the polymer brush as a multilayer structure [37, 38]. However, the expected change in the vdW interactions can be approximated if we assume that the polymer brush is homogeneous, i.e. its properties are constant in the z-direction.

The calculation of the vdW interactions was done following the procedure of Ref. [35] for the three configurations shown in Figure S.6. We used the volume fraction profile determined experimentally for a PVA with similar characteristics as the polymer examined in our study adsorbed on polystyrene particles [36] together with the refractive index and dielectric constant values reported in the literature for PVA solutions [39, 40].

TABLE S.2. Molecular characteristics of the PVAs studied in literature and the obtained decaying lengths

Ref.	Molecular weight [g/mol]	VAc content [%]	Distribution	λ [nm]
Lyklema and van Vliet [17]	42,500[a]	12	Blocky (n_{VAc} unknown)	20
	143,000[a]	12	Blocky ($n_{VAc} = 1.3$) [33])	22
	149,000[a]	16.6	Random	46[c]
Espert *et al.* [18]	10,000[b]	20	Random	6
	55,000[b]	12	Random	9
	155,000[b]	12	Random	16
Sonntag *et al.* [19]	55,000	12	Unknown	10
	123,000	12	Unknown	6
Our study	63,548[b]	8[d]	Blocky ($n_{VAc} = 2$)[d]	18

[a] Viscosity-average molecular weight. For commercial PVA grades it lies slightly below the weight-average values [34].

[b] Weight-average molecular weight.

[c] Unrealistic value as the film most likely had not reached equilibrium. The 55-60 Pa pressure range that was examined and the 9 nm thickness changes that were observed are within experimental error (Fig. S.4).

[d] As determined by ^{13}C NMR spectroscopy. The VAc content from ^{1}H NMR is 12%.

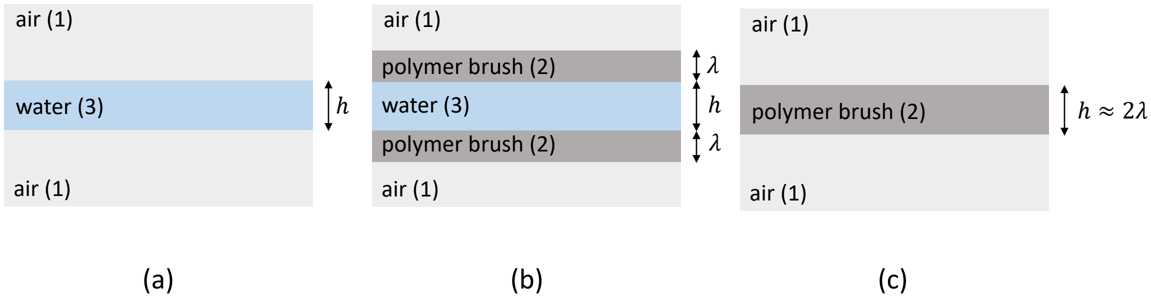

Fig. S 6. **The three different idealized film configurations examined:** (a) The typically employed pure water film, (b) a polymer brush adsorbed at the air/liquid interface, and (c) two overlapping polymer brushes in air.

The calculated Π_{vW} for the film with and without the adsorbed polymer is shown in Figure S.7 with a dashed and solid line, respectively. To exaggerate the effect of the polymer brush, we used

the maximum value for the dielectric constant and the refractive index (instead of e.g. the average one), thus assuming that the volume fraction of the brush has a constant maximum value of 0.5 along its whole 17.8 nm length (attaining thus the maximum values possible from Cosgrove *et al.* [36]).

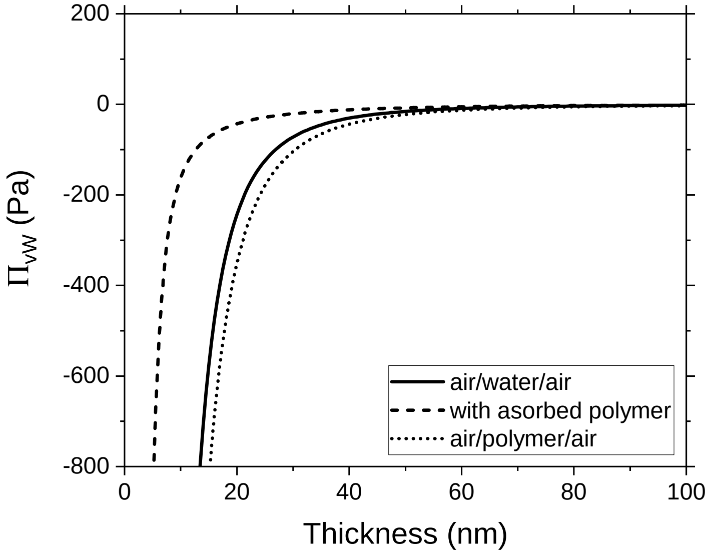

Fig. S 7. **The contribution of vdW interactions in the disjoining pressure:**A pure water film in air (solid line), a water film with a polymer brush in air (dashed line), and a polymer solution film in air (dotted line).

For a given separation distance *h*, the Π_{vW} with adsorbed polymer (dashed line) is smaller than that of a pure water film (solid line), in agreement with the results of Dagastine *et al.* [38]. For this calculation we assume that the separation distance starts at the end of the polymer brush as in Ref. [38].

If it is assumed that the interface is located exactly at the air/polymer interface (Figure S.6c), then the Π_{vW} would be stronger for a given distance, following the results of Bevan and Prieve [41] as the properties of the polymer brush (or solution) dominate the properties of the aqueous film. In this case the vdW interactions are shown with the dotted line in Figure S. 7.

The effect of the different definitions of Π_{vW} on λ is however small. Refitting the Π_{st}, results in $\lambda = 19.2$ nm when the location of the interface is assumed to be at the edge of the polymer brush (Figure S.6b) or $\lambda = 16.6$ nm if the interface is assumed to be at the air/polymer side (Figure S.6c). Therefore, the deviation in λ from the fitted value of 17.8 nm determined for a pure water film is less than the ± 2 nm resolution of microinterferometry and can thus be neglected. Differences are however expected to increase significantly as thickness decreases and the choice of the location

of the interface can be important if the thickness of the film is smaller than 20 nm. Disregarding the vdW interactions, as done for example in Ref. [18] results in $\lambda = 20.5$ nm but completely overpredicts the critical disjoining pressure for film rupture (as vdW interactions are crucial for the film becoming unstable).

4. *Drainage of PVA films*

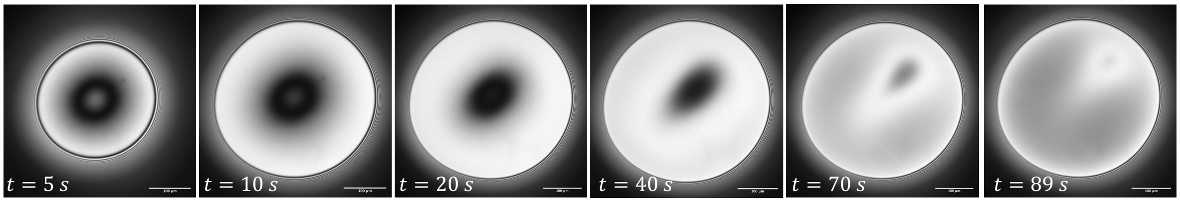

Fig. S 8. **Drainage of PVA films:** A sequence of images of a PVA-stabilized film draining at $\Delta P = 200$ Pa. Drainage proceeds asymmetrically and the dimple becomes unstable and gradually moves towards the rim of the film. At $t = 89$ s rupture occurs. Scalebar is 100 μm.

B. BrijO10 films

As mentioned in the main article, the strata in the BrijO10 that are typically observed due to the layering of micelles inside the film were in our case unstable, as the critical pressure needed for their expulsion was lower than the Laplace pressure due to the curvature of the bike-wheel's hole. Nevertheless, these unstable points can still be observed in draining films and result in a step-wise thinning known as stratification [42]. A thickness profile of a BrijO10 film draining under an application of an extra pressure of 50 Pa is shown in Fig. S.9. Various thickness steps can be observed each corresponding to a different number of contained micelle layers. The length of two opposing BrijO10 molecules was calculated based on the length of the bonds [43]. The length of each ethylene oxide unit is 0.19 nm [44] (with a total of 10 such units in a BrijO10 molecule). The length of the aliphatic chain was calculated from the formula of Tanford [7, 45] and was equal to 2.43 nm.

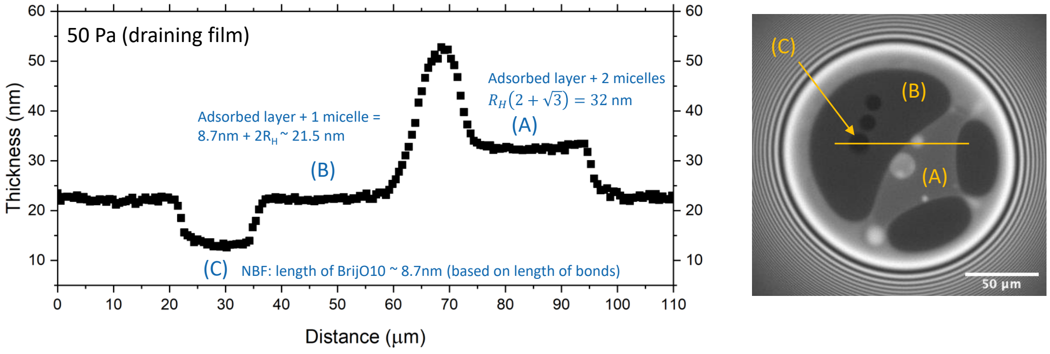

Fig. S 9. **Stratification in a draining BrijO10 film:** *Right.* Microinterferometry image of a BrijO10 film draining under the application of an extra 50 Pa. Three regions with different gray-scale intensity values and thus different thicknesses are indicated. *Left.* The thickness profile that corresponds to the dashed line. The thickness estimation of the three different regions is also shown.

S V. GPC

Gel permeation chromatography (GPC) analyses were performed on a Viscotek GPCmax system equipped with a TDA 302 triple detector array (both Malvern). The eluent was $H_2O+NaNO_3$ at a flow rate of 0.7 $mL{\cdot}\mathrm{min}^{-1}$. Samples were dissolved in the eluent at a concentration of 2 mg·ml^{-1} and passed through a 0.45 μm nylon filter prior injection. The system was calibrated using near monodisperse PMMA standards (Polymer Standard Services).

S VI. NMR RESULTS

The ^{1}H and ^{13}C NMR spectra of PVA are shown in Fig. S.10 a and b, respectively. The determination of the average distribution of VAc units per hydrophobic segment was done using the method of Ref. [46].

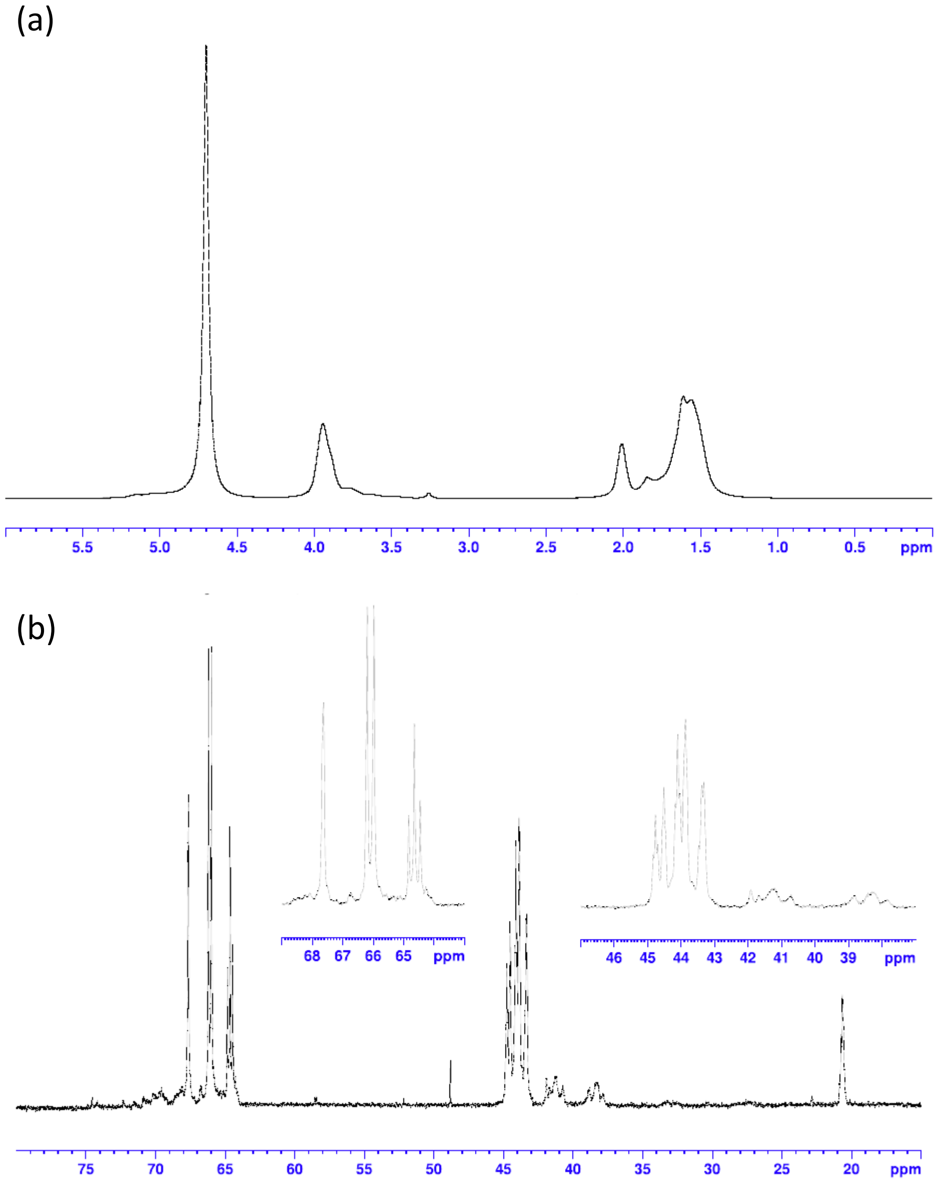

Fig. S 10. **NMR results:** (a) ^{1}H and (b) ^{13}C NMR spectra of the PVA. Details on the experimental procedure can be found in the methods section of the main article.

S VII. SUPPLEMENTARY DATA ON FOAM DRAINAGE

A. Evolution of Bq number with time during foam drainage

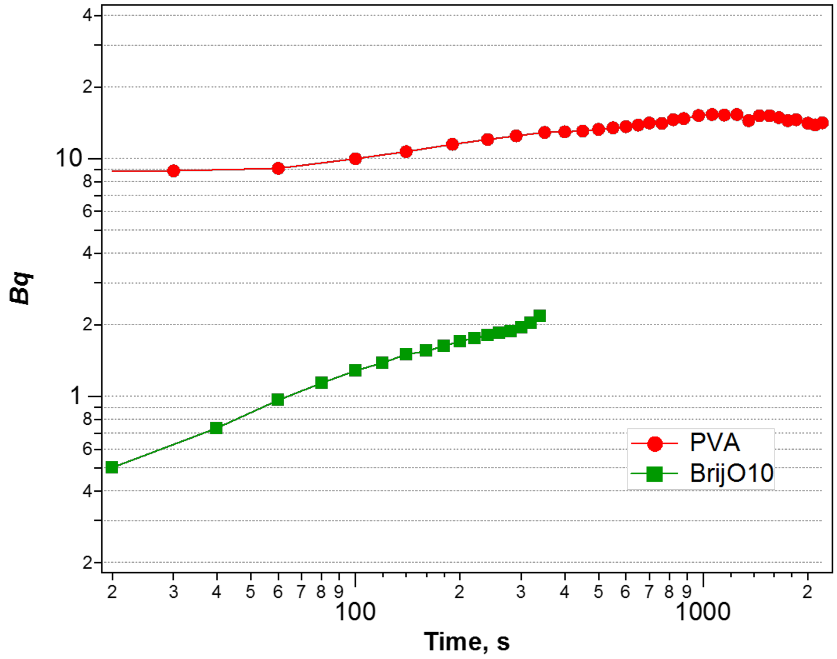

Fig. S 11. **Evolution of Bq number during the foam drainage:** PVA-stabilized foam (red circles) and BrijO10-stabilized foam (green squares). The number is calculated as $Bq = \frac{\eta_s}{\eta r_{PB}}$ with r_{PB} estimated with Kelvin cell model.

B. Film contribution to the foam drainage

In order to assess if the films contribute to the overall film drainage, we calculated the liquid fraction entrapped in the films at the beginning and end of our measurements following the procedure described in Carrier *et al.* [47] using the experimental total liquid fractions, average bubble sizes (determined from the macroscopic foam drainage experiments) and film thickness (from the TFB experiments).

Based on [47] the liquid entrapped in the films is equal to:

$$\phi_f = \frac{0.5hA_f}{V_k} \tag{S.4}$$

Where h is the thickness of the film, $A_f \approx 27L_{PB}^2(1-1.52\sqrt{\phi})^2$ is the average surface area occupied by the films on a bubble (ϕ is the total liquid fraction in the foam), and $V_k = 8\sqrt{2}L^3$ is the volume of a Kelvin cell. $L_{PB} = 2R_b/2.7$ is the length of the Plateau border, and L is the total length of a Plateau border plus a quarter of a node at each end (defined as "dogbone" in [47]. If we assume that $L \approx L_{PB}$, then we can calculate ϕ_f. The input parameters and the calculated liquid fraction of the films are shown in Table S.3.

TABLE S.3. Calculation of liquid fraction in the films

System	Time, s	R_b, μm	h, m	ϕ	ϕ_f	$\frac{\phi_f}{\phi}\cdot 100\%$
BrijO10	0	335	$2{\cdot}10^{-6}$ (assumed)	0.1	$2.6{\cdot}10^{-3}$	2.6%
	340[a]	586	10^{-8}	0.0019	$2.4{\cdot}10^{-5}$	1.3%
PVA	0	156	$2{\cdot}10^{-6}$ (assumed)	0.1	$5.6{\cdot}10^{-3}$	5.6%
	1060[b]	355	$4.5{\cdot}10^{-8}$	0.01	$1.5{\cdot}10^{-4}$	1.5%

[a] Foam collapse by front propagation
[b] Homogeneous collapse

One should note that all the assumptions on the geometrical parameters applies for very dry foams with liquid fractions below 0.02. Therefore, the values for time of 0 s are not strictly correct. For wet foams, the length of PBs are smaller, the size of vertices is more important and, therefore, their contribution to the drainage is considered to be dominant (see S. J. Cox *et al.* [48]). Even if we expect the film thickness to be larger at this stage, the area of films is significantly smaller [49], so that the film contribution to the drainage cannot be very important.

The calculations for the front propagation in PVA foam are also quite approximate since the polydispersity is really important at this stage, and the model of Kelvin cell is not applicable. But even for such big bubbles in a monodisperse foam, the ratio ϕ_f/ϕ would be around 6%.

S VIII. SURFACE TENSION ISOTHERMS

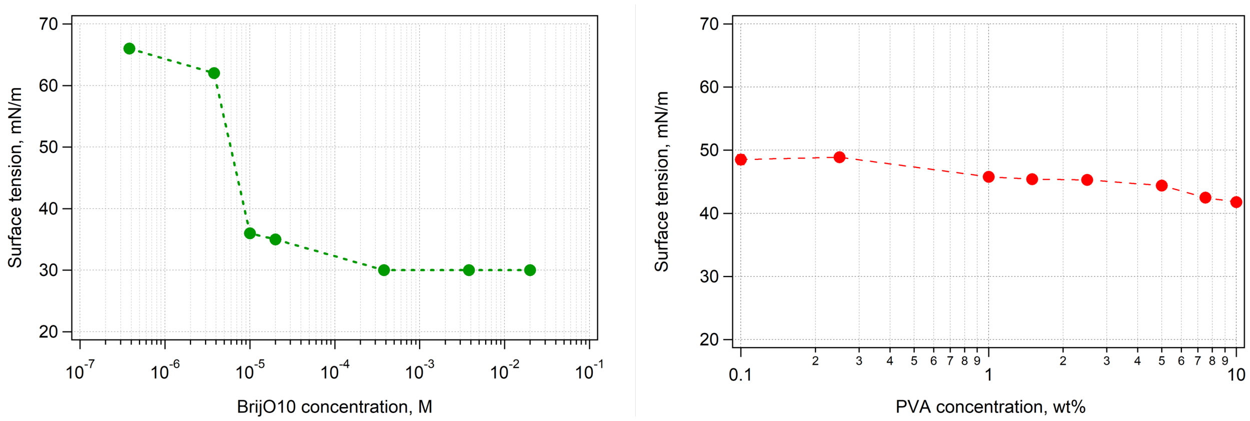

Fig. S 12. **Surface tension isotherms for BrijO10 and PVA:** Surface tension as a function of bulk concentration. The error bars are of the size of the symbols, the lines are guides for the eyes.

S IX. DRAINAGE MOVIES

Movie S1: Drainage of a PVA-stabilized film at $\Delta P = 50$ Pa. The film drains symmetrically until it reaches its equilibrium constant thickness (Speed $5\times$).

Movie S2: Drainage of a BrijO10-stabilized film at $\Delta P = 50$ Pa. The film shows stratification until it reaches its equilibrium constant thickness (Speed $1\times$).

Movie S3: Drainage of a PVA-stabilized film at $\Delta P = 200$ Pa. The film shows asymmetric drainage until it ruptures (Speed $1\times$).

REFERENCES

[1] C. F. Brooks, G. G. Fuller, C. W. Frank, and C. R. Robertson, Langmuir **15**, 2450 (1999).

[2] S. Reynaert, C. F. Brooks, P. Moldenaers, J. Vermant, and G. G. Fuller, Journal of Rheology **52**, 261 (2008).

[3] D. Renggli, A. Alicke, R. H. Ewoldt, and J. Vermant, Journal of Rheology **64**, 141 (2020), https://doi.org/10.1122/1.5130620.

[4] T. Verwijlen, P. Moldenaers, H. A. Stone, and J. Vermant, Langmuir **27**, 9345 (2011).

[5] J. Tajuelo, J. M. Pastor, F. Martínez-Pedrero, M. Vázquez, F. Ortega, R. G. Rubio, and M. A. Rubio, Langmuir **31**, 1410 (2015).

[6] E. M. Duyvis, *The equilibrium thickness of free liquid films* (University of Utrecht, 1962).

[7] C. Tanford, *The hydrophobic effect: formation of micelles and biological membranes 2d ed* (J. Wiley., 1980).

[8] C. Stubenrauch, J. Schlarmann, and R. Strey, Physical Chemistry Chemical Physics **4**, 4504 (2002).

[9] C. Stubenrauch, O. J. Rojas, J. Schlarmann, and P. M. Claesson, Langmuir **20**, 4977 (2004).

[10] A. F. Gallaugher and H. Hibbert, Journal of the American Chemical Society **58**, 813 (1936).

[11] M. Dover and W. Hensley, Industrial & Engineering Chemistry **27**, 337 (1935).

[12] T. Cosgrove, T. G. Heath, K. Ryan, and T. L. Crowley, Macromolecules **20**, 2879 (1987).

[13] T. van Vliet, *Interactions between adsorbed macromolecules: measurements on emulsions and liquid films* (Wageningen University and Research, 1977).

[14] P. De Gennes and P. Pincus, Journal de Physique Lettres **44**, 241 (1983).

[15] L. L. Olijve, M. M. Hendrix, and I. K. Voets, Macromolecular Chemistry and Physics **217**, 951 (2016).

[16] G. Fleer, M. C. Stuart, J. M. Scheutjens, T. Cosgrove, and B. Vincent, *Polymers at interfaces* (Springer Science & Business Media, 1993).

[17] J. Lyklema and T. Van Vliet, Faraday Discussions of the Chemical Society **65**, 25 (1978).

[18] A. Espert, P. Omarjee, J. Bibette, F. L. Calderon, and O. Mondain-Monval, Macromolecules **31**, 7023 (1998).

[19] H. Sonntag, B. Ehmke, R. Miller, and L. Knapschinski, Advances in Colloid and Interface Science **16**, 381 (1982).

[20] O. Mondain-Monval, A. Espert, P. Omarjee, J. Bibette, F. Leal-Calderon, J. Philip, and J.-F. Joanny, Physical Review Letters **80**, 1778 (1998).

[21] J. Philip, T. Jaykumar, P. Kalyanasundaram, B. Raj, and O. Mondain-Monval, Physical Review E **66**, 011406 (2002).

[22] J. Philip, G. Gnanaprakash, T. Jayakumar, P. Kalyanasundaram, and B. Raj, Macromolecules **36**, 9230 (2003).

[23] A. S. Alkindi, Y. M. Al-Wahaibi, and A. H. Muggeridge, Journal of Chemical & Engineering Data **53**, 2793 (2008).

[24] G. Johari and E. Whalley, in *Faraday Symposia of the Chemical Society*, Vol. 6 (Royal Society of Chemistry, 1972) pp. 23–41.

[25] V. Bergeron, Journal of Physics: Condensed Matter **11**, R215 (1999).

[26] W. W. Graessley, Polymer **21**, 258 (1980).

[27] T. Canal and N. A. Peppas, Journal of biomedical materials research **23**, 1183 (1989).

[28] C. M. Kok and A. Rudin, Die Makromolekulare Chemie, Rapid Communications **2**, 655 (1981).

[29] P.-D. Hong, C.-M. Chou, and C.-H. He, Polymer **42**, 6105 (2001).

[30] G. Fleer, J. Van Male, and A. Johner, Macromolecules **32**, 825 (1999).

[31] A. Semenov, J. Bonet-Avalos, A. Johner, and J. Joanny, Macromolecules **29**, 2179 (1996).

[32] R. Owen, J. Crocker, R. Verma, and A. Yodh, Physical Review E **64**, 011401 (2001).

[33] B. Budhlall, K. Landfester, E. Sudol, V. Dimonie, A. Klein, and M. El-Aasser, Macromolecules **36**, 9477 (2003).

[34] L. Koopal, Colloid and Polymer Science **259**, 490 (1981).

[35] J. N. Israelachvili, *Intermolecular and surface forces* (Academic press, 2015).

[36] T. Cosgrove, T. L. Crowley, K. Ryan, and J. R. Webster, Colloids and surfaces **51**, 255 (1990).

[37] B. Ninham and V. Parsegian, The Journal of Chemical Physics **53**, 3398 (1970).

[38] R. R. Dagastine, M. Bevan, L. R. White, and D. C. Prieve, The Journal of Adhesion **80**, 365 (2004).

[39] C. M. Atkinson, R. Dietz, and M. A. Francis, Polymer **21**, 891 (1980).

[40] R. J. Sengwa and K. Kaur, Polymer international **49**, 1314 (2000).

[41] M. A. Bevan and D. C. Prieve, Langmuir **16**, 9274 (2000).

[42] V. Bergeron and C. Radke, Langmuir **8**, 3020 (1992).

[43] E. S. Basheva, P. A. Kralchevsky, K. D. Danov, K. P. Ananthapadmanabhan, and A. Lips, Physical Chemistry Chemical Physics **9**, 5183 (2007).

[44] M. Rosch, by MJ Schick, Marcel Dekker, New York, NY , 753 (1967).

[45] R. Rajagopalan and P. C. Hiemenz, Marcel Dekker, New-York **8247**, 369 (1997).

[46] T. Moritani and Y. Fujiwara, Macromolecules **10**, 532 (1977).

[47] V. Carrier, S. Destouesse, and A. Colin, Physical Review E **65**, 061404 (2002).

[48] S. Cox, G. Bradley, S. Hutzler, and D. Weaire, Journal of Physics: Condensed Matter **13**, 4863 (2001).

[49] A. M. Kraynik and D. Reinelt, *Structure and rheology of wet foam.*, Tech. Rep. (Sandia National Lab.(SNL-NM), Albuquerque, NM (United States), 2008).
